# Development and Biomedical Application of Non-Noble Metal Nanomaterials in SERS

**DOI:** 10.3390/nano14201654

**Published:** 2024-10-15

**Authors:** Liping Chen, Hao Liu, Jiacheng Gao, Jiaxuan Wang, Zhihan Jin, Ming Lv, Shancheng Yan

**Affiliations:** 1 School of Chemistry and Life Sciences, Nanjing University of Posts and Telecommunications, Nanjing 210023, China; 18061659916@163.com; 2 School of Integrated Circuit Science and Engineering, Nanjing University of Posts and Telecommunications, Nanjing 210023, China; 2023221102@njupt.edu.cn (H.L.); 1223228118@njupt.edu.cn (Z.J.); 3 School of Science, Nanjing University of Posts and Telecommunications, Nanjing 210023, China; 2023080906@njupt.edu.cn; 4 School of Materials Science and Engineering, Nanjing University of Posts and Telecommunications, Nanjing 210023, China; 2022060706@njupt.edu.cn; 5 Department of Medical Engineering, Medical Supplies Center of PLA General Hospital, Beijing 100039, China; yxgcbj@163.com

**Keywords:** surface-enhanced Raman scattering, biomedical marker detection, non-noble metals, enhancement factor

## Abstract

Surface-enhanced Raman scattering (SERS) is vital in many fields because of its high sensitivity, fast response, and fingerprint effect. The surface-enhanced Raman mechanisms are generally electromagnetic enhancement (EM), which is mainly based on noble metals (Au, Ag, etc.), and chemical enhancement (CM). With more and more studies on CM mechanism in recent years, non-noble metal nanomaterial SERS substrates gradually became widely researched and applied due to their superior economy, stability, selectivity, and biocompatibility compared to noble metal. In addition, non-noble metal substrates also provide an ideal new platform for SERS technology to probe the mechanism of biomolecules. In this paper, we review the applications of non-noble metal nanomaterials in SERS detection for biomedical engineering in recent years. Firstly, we introduce the development of some more common non-noble metal SERS substrates and discuss their properties and enhancement mechanisms. Subsequently, we focus on the progress of the application of SERS detection of non-noble metal nanomaterials, such as analysis of biomarkers and the detection of some contaminants. Finally, we look forward to the future research process of non-noble metal substrate nanomaterials for biomedicine, which may draw more attention to the biosensor applications of non-noble metal nanomaterial-based SERS substrates.

## 1. Introduction

At the end of the last century, scientists became intrigued by the significant changes observed in the Raman spectrum of pyridine molecules when positioned near a silver electrode [1]. Electrochemical experiments showed that pyridine adsorbed on a rough silver electrode had a high signal-to-noise ratio and high-intensity Raman spectrum. Henceforth, the study of surface-enhanced Raman spectroscopy (SERS) was rapidly developed in the past decades. Due to its features of rapid detection, trace-free measurement, and fingerprint correspondence, SERS is widely used in bioanalysis, chemical identification, environmental protection, food safety, etc., and it is regarded as a promising technique for microanalysis [2,3].

Raman enhancement factor (EF) is a generic term used to describe how many orders of magnitude increase in signal is expected under SERS conditions using a nanostructured substrate compared to an “equivalent” Raman experiment without a substrate [4]. EF is a metric that quantifies the extent of Raman signal amplification for a molecule achieved through the surface-enhanced Raman scattering technique. The following equation can calculate it:

(1) $$\mathrm{EF}=\frac{I_{\mathrm{SERS}}}{I_{\mathrm{bulk}}}\frac{N_{\mathrm{bulk}}}{N_{\mathrm{SERS}}}.$$

The calculation of EF involves two essential ratios: I_SERS_/I_bulk_ (signal enhancement) and N_SERS_/N_bulk_ (contact efficiency), where I_SERS_ represents the intensity of the enhanced Raman signal and I_bulk_ represents the classical Raman signal obtained in the absence of the SERS substrate, N_SERS_ the number of molecules excited in SERS, and N_bulk_ the number of molecules excited in classical Raman [5]. This amplification of the molecular Raman signal is predominantly achieved via interaction with the metallic surface. The accurate calculation of the EF is related to the nature of the nanostructured SERS-active substrate, including the composition, size, shape, structure, local environment, surface chemistry, and interactions with the target molecules, among other factors. EF is apparent in evaluating the enhancement effect of the SERS technique on molecular signals under specific conditions and serves as a reference index for comparing different experimental conditions and materials.

According to current research reports, noble metal nanomaterials are regularly used as SERS substrates due to their high free carrier density and strong localized surface plasmon resonance (LSPR). This condition triggers a significant LSPR when exposed to electromagnetic radiation, subsequently creating a considerable electromagnetic field in the surrounding area, thus substantially enhancing target molecules’ target molecular Raman signals and realizing ultra-high SERS sensitivity [6,7]. In this mechanism, metallic nanoparticles or nanostructures can absorb incident light and excite the surface plasmon resonance on the surface [8,9]. Resonant fields enhance the molecular Raman signal, with metal nanostructure properties crucially affecting enhancement, such as the shape, size, and arrangement of the metal nanostructures. However, considering its high price and shortcomings in selectivity, accuracy, and stability, noble metal nanomaterials are subject to many limitations when put into real production.

In contrast, the nanomaterials based on chemical enhancement of non-noble metals will overcome these shortcomings. CM enhances molecular Raman signals through chemical interactions between molecules and metal surfaces. Changes in electron density and charge transfer on the metal surface enhance Raman scattering. Chemical enhancement, affected by molecule adsorption sites, structures, and metal surface properties, can significantly boost Raman signals through bond formation or charge transfer. For example, the semiconductors such as MoO_2_, GaP, WO_3_, etc., transformed into metal-like plasmon materials by doping or the introduced defects, exhibited enhanced properties comparable to noble metal nanomaterials, in which interfacial charge transfer (ICT) plays a dominant role. They not only achieve efficient electromagnetic enhancement with a low damping rate and reduced Ohmic loss [10], but also their high selectivity can distinguish specific molecules from complex environments. In addition, the excitation wavelength of SERS is usually in the feasible near-infrared region, which is less damaging to biomaterials. Due to the reduced photothermal conversion, non-noble metal systems are more highly durable and less susceptible to photothermal degradation by equipartition excitation resonance. It is worth emphasizing that this advantage is crucial in biomedical applications. Therefore, non-noble metal SERS are used in various fields such as single molecule detection, biochemistry, and environmental pollution [11,12].

For practical biomedical applications, SERS’s single-molecule (SM) detection generally requires an EF greater than 10^13^ and a detection limit better than 1 femtomole (fM). This detection intensity is related to the design of the substrate, the substrate material [13], the chemical pretreatment, and other factors. The physicochemical properties of non-noble nanomaterials, such as exciton Bohr radius, energy band structure, and electron density, can be easily tuned, making them desirable SERS substrate materials. The research and development of non-noble metal substrates greatly expanded the application scope of SERS, leading to significant advances in SERS detection technology [14]. In recent years, researchers successfully prepared novel SERS substrates based on non-noble metal nanomaterials. SERS substrates can be broadly categorized into two strategies, one is based entirely on non-noble metal systems, such as carbon nanomaterials, metal oxides, metal-organic frameworks (MOFs), and transition metal carbides and nitrides (MXenes) [15,16]. Another type is the hybrid noble/non-noble metal systems such as Au NPs/CNT, CNF-Cu_2_O/Ag, Cu_2_O/Ag_x_, and AuNP dimer/MXenes. The combination of plasmon materials (Au, Ag, etc.) and non-plasmon materials was improved into new types of plasmon materials to create more generalized and improved well metal-framed substrates. As a result, some innovative non-noble metal strategies were proposed to realize biosensing substrates with ultra-high SERS enhancement [17,18], which is expected to be a new approach for SERS detection.

**Figure 1:**
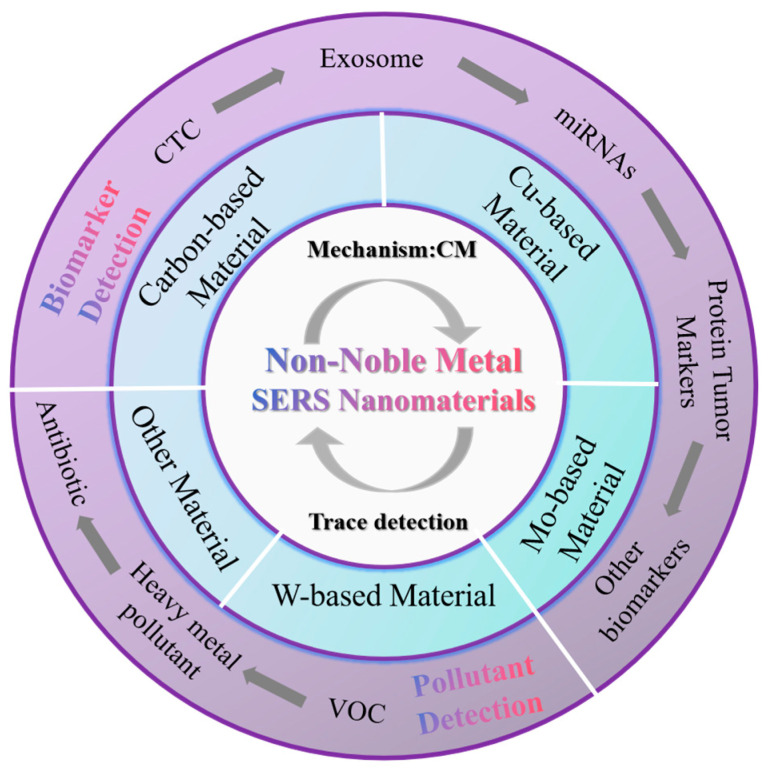
Overview of the development of non-noble metal nanomaterials in SERS.

As the demand for biomedical molecular detection increased, SERS technology evolved to allow for fast, simple and efficient detection. Non-noble metal-based biosensors and a variety of medical testing devices came into the limelight, which are more economical and harmless than noble metals. In this paper, we review several major non-noble metal Raman substrate materials in recent years and discuss their applications in analytical applications such as biomarker detection, contaminant detection, etc., which will help to enhance the understanding of non-noble metal substrates for bioanalysis (Figure 1).

## 2. Non-Noble Metal SERS Nanomaterials

### 2.1. C-Based Nanomaterials

**Figure 2:**
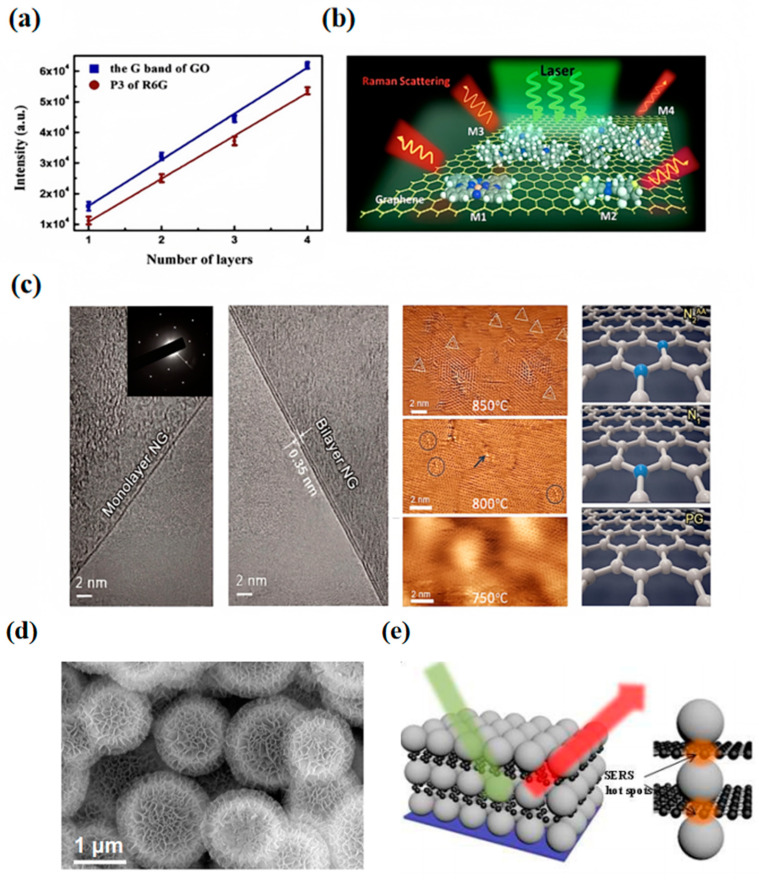
(**a**) Curves of g-band Raman intensity (blue) and P3 peak of R6G (red) with the number of layers of graphene oxide flakes [19]. (**b**) The schematic of GERS molecular selectivity shows different types of M1, M2, M3, and M4 [22]. (**c**) HRTEM and STM images of NG after transfer and typical STM images of NG sheets synthesized with different doping parameters [25]. The triangles and the circles here indicate the STM double N substitution configuration, and the arrow indicates the STM single N substitution. (**d**) SEM image of graphdiyne hollow microspheres [29]. (**e**) Schematic of the 3D structure of three-layer silver nanoparticles [33].

Graphene has the advantages of high enhancement factor, wide frequency response range, modulation performance, durability, and stability, as well as environmental friendliness, which makes it one of the materials that attracted much attention in SERS, and its Raman-enhanced effect is usually called graphene-enhanced Raman scattering (GERS). Graphene is also an efficient metal substrate for detecting trace organic molecules due to its chemical inertness and good biocompatibility. Retracing the earlier studies on the enhancement mechanism of graphene, Yang et al. [19] systematically investigated SERS on graphene, graphene oxide (GO), and reduced graphene oxide (r-GO) using R6G as a probe molecule and found that the Raman spectra on the three SERS substrates are significantly different due to the local chemistry of substrate groups and the global π-conjugation network with different enhancement contributions. In general, the Raman signals of the molecules increase with the number of graphene oxide layers, and the oxidation groups may play an important role (Figure 2a). Differently, Ling et al. [20,21] showed that the GERS chemical enhancement is highly sensitive to molecular orientation. Diverse molecular orientations result in varying levels of molecular interaction with graphene. The greater this interaction, the more pronounced the enhancement of the Raman signal becomes, a phenomenon known as the “first-layer effect”.

Subsequently, to explain the GERS mechanism on different neutral molecules, Huang et al. [22] focused on molecules with large Raman cross-sections and investigated the mechanisms of molecular selectivity in GERS measurements for different applications. They examined three different types of molecules separately. (1) encompasses molecules with similar molecular structures but different energy levels and includes different phthalocyanine (Pc) derivatives: copper phthalocyanine (CuPc) and zinc phthalocyanine (ZnPc); (2) involves molecules with similar energy levels but different molecular structures, such as tetrathienophenazine (TTP) and tris(4-carbazoyl-9-ylphenyl) amine (TCTA); and (3) includes other molecules of interest, such as 3,5-tris(N-phenylbenzimiazole-2-yl) benzene (TPBi), bathocuproine (BCP), and so forth. GERS predominantly operates via a chemical mechanism, thereby showcasing a distinctive molecular selectivity (Figure 2b). First, they determined that the molecules should have a suitable HOMO/LUMO energy level arrangement. The highest occupied molecular orbital (HOMO) and the lowest unoccupied molecular orbital (LUMO) energies are in the right range relative to the Fermi energy levels of graphene. This is a basic requirement for molecule–graphene structure compatibility and interaction. Second, the molecular structure must have appropriate point group symmetry, including D_nh_ symmetry, which is an additional requirement for molecule–graphene structural compatibility, resulting in stronger charge transfer and greater GERS EF.

Interestingly, Marumi et al. [23] further developed a simple and controllable n-doping method to prepare carbon-based SERS substrates. They systematically investigated the chemical and structural changes of electrostatically spun polyacrylonitrile (PAN) nanonets carbonized at different temperatures, and demonstrated how graphite N doping can be performed on carbonized PAN-based SERS substrates at 1000–1200 °C for the first time. Due to the interaction between two competing reactions, the expansion of the conjugated system and the N doping effect, the PAN-based substrate reaches an optimal equilibrium when carbonized at 1200 °C in a low vacuum environment (0.2 Pa), an approach that facilitates the application of sensors.

Recently, a novel 3D graphene oxide aerogel (GOA) was constructed by hydrothermal reactive self-assembly [24], followed by freeze-drying of graphene oxide, and then used as an ultrasensitive SERS substrate. Due to its large specific surface area favorable for molecular adsorption, the optimized GOA exhibited a high SERS EF (3.1 × 10^4^) and a low detection limit (10^−8^ M) for dye molecules. Feng et al. [25] took advantage of the differences in nitrogen doping to control the displacement of the Fermi energy levels of graphene and utilized the enhanced Raman scattering of N-doped graphene to prepare ultra-sensitive graphene. They use enhanced Raman scattering from N-doped graphene to prepare an ultrasensitive molecular sensor. If this displacement aligns with the molecule’s LUMO, charge transfer is augmented, thereby significantly amplifying the vibrational Raman modes of the molecule (Figure 2c).

Recent findings showed that the number of carbon layers, oxidation states, and surface groups are closely related to the SERS properties of carbon substrates. Graphdiyne (GDY) is a well-known two-dimensional carbon allotrope [26], which consists of a benzene ring portion (sp^2^ carbon atoms), butadiene (sp carbon atoms) linkers, and well-dispersed electron-rich cavities to form a large π-connected structure, GDY is the first carbon material with a two-dimensional fast-transfer channel for electrons and a three-dimensional fast transfer channel for ions [27]. Zhang et al. [28] reported a surface-based microemulsion method based on surface-active agents to synthesize GDY hollow microspheres. Considering the interference of surfactants with various physicochemical processes, the potential mechanisms of surfactant-free synthesis and Raman enhancement need to be further explored. Therefore, their team further synthesized GDY hierarchical hollow microspheres [29] with self-supporting structure and ultra-high specific surface area by a surfactant-free liquid–liquid interface-induced growth method (Figure 2d). By integrating first principles density functional theory simulations with practical experimentation, it is found that the potential effect behind this SERS phenomenon is mainly affected by strong interfacial interactions within the graphene–molecule system and that the substrate shows a strong SERS effect, with an EF of 3.7 × 10^7^. It has a limit of detection of R6G of 1 × 10^−12^ M, approximately 1000 times that of graphene.

In addition, to improve the substrate’s SERS performance, doping with noble metals or optimizing the morphology for nanomaterials substrates can achieve significant results. Such as a highly efficient SERS substrate using silver nanoparticle-modified GO composites [30]. Materials characterization was conducted utilizing X-ray diffraction, transmission electron microscopy, and scanning electron microscopy techniques. All experimental results show that the developed Ag@GO composite substrate exhibited significant enhancement. Similarly, Chulsoo Kim et al. [31] employed carbon nanowalls (CNWs) as main nanostructures, increased the hotspot density by oxygen plasma, and embedded silver nanoparticles (Ag NPs) to obtain robust and stable SERS substrates. The large specific surface area of CNWs and the graphene domains provided, respectively, the dense hotspots and high charge mobility, and the analysis confirmed a proportional increase in Raman signal intensity with increasing R6G concentration (10^−6^ M to 10^−10^ M). More, gold nanoparticles were uniformly deposited on carbon-based substrates (CNT sheets) using one-step wet chemistry, magnetron sputtering, and so on [32]. The modified flexible nanomaterials based on Au NPs/CNT has an enhancement effect 1000 times higher than the previous substrate.

In addition, Li et al. [33] synthesized three-dimensional SERS nanomaterial with different numbers of silver nanoparticles (Ag NPs) layers, they applied multilayered GO as a spacer layer to investigate the layer effect of graphene and the influence of the number of doped noble metal particles on the SERS enhancement. It was observed that the SERS effect showed an increasing trend with the growth of the number of Ag NPs stacked layers and saturated when more than four layers were reached (Figure 2e). Figure 2e schematically exhibits a fabricated 3D Ag NPs structure and the hot spots mainly exist on the nanogap region created by GO between the two Ag NPs layers. This study reveals that the optimization of the SERS performance of 3D nanostructures mainly originates from the hotspot regions formed in the top layers, which are closely related to the distribution and interactions of Ag NPs in each layer inside the 3D structures. Furthermore, this study comprehensively evaluated 3D nanostructures assembled from varying quantities of silver nanoparticle layers, utilizing finite-difference time-domain (FDTD) simulations. The fabricated 3D SERS substrates demonstrated remarkable sensitivity, capable of efficiently identifying R6G and crystalline violet (CV) with detection thresholds as low as 10^−15^ M and 10^−12^ M, respectively, realizing ultra-sensitive detection.

### 2.2. Cu-Based Nanomaterials

As a transition metal, Cu has specific chemical stability and catalytic activity. It can be used in the broader range of excitation light due to its negligible absorption of specific wavelengths, which is helpful for practical SERS measurements of different Raman-active substances. The SERS effect excited by the generation of surface plasmon resonance from a single metal Cu is well known, but the CM mechanism based on Cu_2_O is increasingly studied. However, Cu substrates and Cu oxides are unstable and require suitable protection measures to prevent rapid oxidation of their surfaces from affecting the SERS performance. Hence, the surface state needs to be finely tuned for practical applications. Cuprous oxide (Cu_2_O) is a p-type semiconducting metal oxide with a direct band gap of 2.17 eV [34], a potential material for catalytic and sensing properties. In recent years, the synthesis of Cu_2_O nanostructures, such as nanoflowers, nanotubes [35], nanoparticles [36,37], polyhedral [38], and microspheres [39] emerged. A synergistic effect occurs during the attachment of Ag NPs onto the Cu_2_O surface, leading to an enhancement of the local electric field. The high charge density produces a strong LSPR enhancement effect.

**Figure 3:**
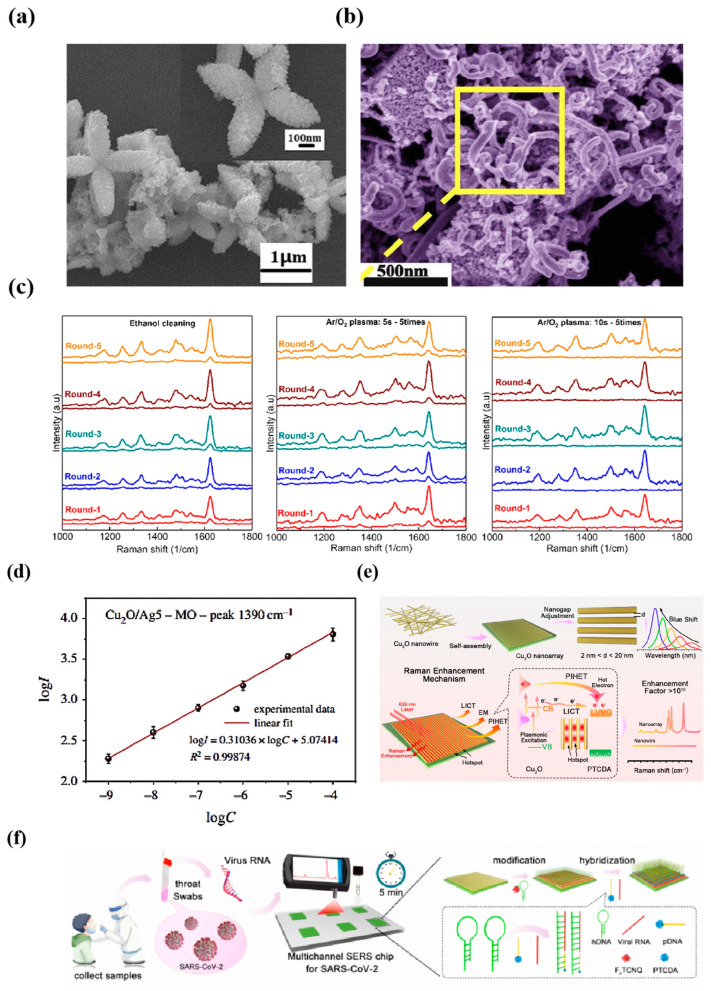
(**a**) Flower-like Cu_2_O@Ag TEM image [42]. (**b**) Typical SEM image of Ag NVs/Cu_2_O heterostructure [44]. (**c**) Substrate rinsed with ethanol 3 times at room temperature, after Ar/O_2_ mixed plasma treatment for 5 s × 5 times, and for 10 s × 5 times [46]. (**d**) The linearity of the logarithm of the 1390 cm^−1^ peak intensity versus the logarithm of the MO concentration and the error bars indicate standard deviation [49]. (**e**) Self-assembly of Cu_2_O nanoarrays with nanogap tuning and Raman enhancement mechanism [50]. (**f**) Schematic of the working principle of the respiratory RNA virus-sensing platform based on Cu_2_O NAs [50].

Surface-enhanced Raman spectroscopy was found to be an excellent technique for the study of thin films as early as the end of the last century, with the first SERS studies of oxides formed on copper surfaces exposed to natural atmospheric conditions being reported [40]. This study lays the foundation for the subsequent development of cuprous oxide for SERS enhancement. Zhang et al. [41] developed a recrystallization-induced self-assembly (RISA) strategy for the construction of three-dimensional cube-like Cu_2_O superstructures, in which three-dimensional cube-like Cu_2_O superstructures were constructed from Cu_2_O mesoporous spheres (MPSs). During the RISA process, many defects are generated due to the lattice fusion of neighboring Cu_2_O MPS. Due to the abundance of defects, new surface states are formed, which promote charge transfer in the semiconductor molecule system and effectively enhance the Raman scattering of the target molecules to solve the problem of low sensitivity. Subsequently, Li et al. [42] synthesized Cu_2_O@Ag-type nanoparticles by attaching silver particles to the surface of Cu_2_O nanoparticles and coated them with a layer of MIP by atom transfer radical polymerization. It was found that the morphology of Cu_2_O was in the form of nanoflower (Figure 3a), and this SERS substrate was reported to be a new method for determining trace chlorophenols in river water; regarding the other way to combine silver and Cu_2_O, Liu et al. [43] prepared silver films with different thicknesses on the copper oxide (Cu_2_O) buffer layer, and the XRD and AFM results show that the Cu_2_O buffer layer had the effect of improving the surface quality and crystallinity of the Cu_2_O-Ag composite film, which was extremely sensitive for the detection of a variety of molecules, such as RhB, methylene blue (MB), and R6G. Interestingly, Jiao et al. [44] constructed a special heterostructure connecting Cu_2_O nano-octahedra with intertwined Ag nanowires (NVs), which intertwine between neighboring Cu_2_O octahedra as an efficient electron transport pathway and are capable of significantly accelerating the effective separation process between electrons and holes, thus effectively suppressing the photogenerated carrier composite phenomenon, and thus substantially enhancing the CM effect of the Cu substrate (Figure 3b). The Ag NVs/Cu_2_O heterostructure exhibits excellent SERS activity, which is about 2.7 and 7.0 times higher than the monodispersed Ag- or Au-nanoparticles-modified Cu_2_O, respectively. The substrate achieved an ultra-low detection limit (CV, 10^−14^ M) with excellent immunity and selectivity.

In addition, Luo et al. [45] synthesized Cu_2_O sub-microcubes under environmentally friendly conditions using 2,2,6,6-tetramethylpyridine-1-oxo (TEMPO) oxidized cellulose nanoprincipal fibers as reducing and stabilizing agents. Silver nanoparticles (Ag NPs) were then modified on the surface of Cu_2_O cubes by substitution reaction. CNF-Cu_2_O/Ag could detect MB down to 10^−8^ M with a maximum EF of 4.0 × 10^4^. Additionally, Vasyl Shvalya et al. [46] grew a CuO/Cu_2_O heterostructure by oxygen flow-assisted thermal annealing method, and constructed a Cu_2_O chestnut-like substrate after activation of 80 nm Au/Pd alloy film with an analytical EF as high as 5 × 10^5^. The substrate still showed excellent robustness after cleaning with ethanol or Ar/O_2_ mixed plasma (Figure 3c), and the excellent signal recovery indicated that the plasmon layer has good stability, and the self-cleaning efficiency is close to 100%. Surface Raman enhancement can detect trace levels of target molecules adsorbed on the surface of metal or semiconductor substrates and prepare hybridized materials between noble metals and semiconductors to detect certain toxic organic dyes. Subsequently, Luo et al. [47] prepared Cu_2_O thin films with three-sided pyramidal and four-sided pyramidal structures, which belonged to (100) and (111) surfaces, respectively, by adjusting the pH of the electrolyte using electrodeposition. When 4-NBT was used as the probe molecule, the EF of Cu_2_O (100) was about 2.7-fold higher than that of Cu_2_O (111), and the detection limit was lower up to 1 × 10 μM. Moreover, the Cu_2_O films exhibited outstanding homogeneity, reproducibility, and stability attributes, characterized by a remarkably low relative standard deviation (RSD) and high sensitivity, rendering them apt for practical applications in real-world settings. Rani et al. [48] constructed a bimetallic nanoparticle sensor by plating silver nanorods (AgNRs) on copper nanoflowers (CuNFs) prepared by both methods using chemical reduction and chemical etching, respectively. The structural properties and purity of the sensors were analyzed through a series of methods. Experiments showed that the AgNRs-deposited CuNFs sensor possessed a significant SERS activity with the highest EF of 10^9^ for the dye molecule R6G and the lowest detection limit of 10^−13^ M. In addition, TTH Pham et al. [49], based on the Cu_2_O microcubes, combined with silver nanoparticles (Ag NPs) to determine the trace level of MO in aqueous solution, a series of Cu_2_O/Ag_x_ (x = 1~5) hybrids with different Ag dosages were prepared by solvothermal and reduction methods, and the highest SERS activity was observed for Cu_2_O/Ag_5_ nanocomposites among the Cu_2_O/Ag heterojunctions formed. The detection limit of Cu_2_O/Ag_5_ for methyl orange (MO) was as low as 1 nM, and the enhanced detection limit was 10 nM. Notably, the MO detection threshold using the Cu_2_O/Ag_5_ substrate was remarkably low at 1 nM with an EF of 4 × 10^8^. Specifically, within the concentration interval spanning from 10^−9^ to 10^−4^ M, a linear correlation was observed between the logarithm of the Raman intensity at 1390 cm^−1^ and the logarithm of the MO concentration, with a robust linear correlation coefficient (R2) of 0.99874 (Figure 3d).

Recently, Feng et al. [50] constructed the first large-area Cu_2_O nanoarrays (NAs) with tunable gaps by utilizing a novel plasmon-induced hot electron transfer (PIHET) mechanism (Figure 3e). The Cu_2_O nanoarrays with tunable gaps were first assembled from Cu_2_O nanowires (Cu_2_O NWs) with copper vacancies (VCu) and oxygen vacancy (Vo) defects; they produced strong LSPR absorption, with a free carrier density of 1.78 × 10^21^ cm^−3^, which is comparable to that of noble metals, and set an EF of 3.19 × 10^10^ in semiconductor substrates. By generating high-energy electrons, the mechanism enhances charge transfer efficiency between the Cu_2_O NAs and the molecules they adsorb, enabling a direct transformation of absorbed light into electrical energy. The thermal electron transfer from Cu_2_O NAs to the molecules explicitly observed in transient absorption (TA) spectra on ultrafast time scales (<1 ps) confirms this PIHET process, which is a new Raman enhancement mechanism never reported in semiconductors. Subsequently, this ultrasensitive and selective Cu_2_O NAs substrate with significant EF was developed to detect trace biological species such as severe acute respiratory syndrome coronavirus 2 (SARS-CoV-2) RNA. The development of this Cu_2_O nanoarray as an enzyme- and amplification-free SERS microarray allowed for the sensitive and rapid quantification of SARS-CoV-2 RNA in less than 5 min, with a limit of detection (LOD) as low as 60 copies/mL. This respiratory RNA virus sensing platform works as shown below (Figure 3f).

### 2.3. Mo-Based Nanomaterials

**Figure 4:**
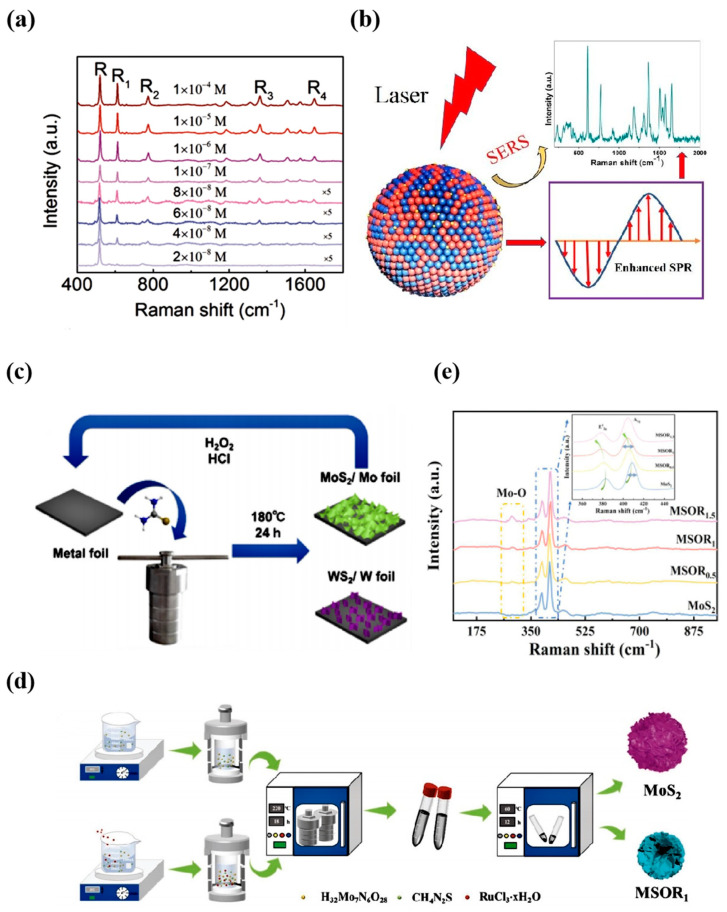
(**a**) Raman spectra of R6G molecules deposited on MoO_2_ substrates after immersion in different concentration solutions [53]. (**b**) Proposed synergistic plasmon enhancement mechanism for Mo/MoO_2_ hybridized SERS substrates [55]. (**c**) Schematic diagram of one-step synthesis of molybdenum disulfide and WS_2_ using metal foil [57]. (**d**) Schematic of MoS_2_ and MSOR_1_ nanoparticle synthesis pathways with different structures [64]. (**e**) Raman spectra of MoS_2_, MSOR_0.5_, MSOR_1_, and MSOR_1.5_ [64].

Among various types of non-noble metal oxides, MoO_2_ has high chemical activity. Modifying and functionalizing surfaces is easy, and it can increase the interaction force with the molecules to be detected by introducing specific functional groups and thus enhancing the SERS effect. Since the first fabrication of molybdenum oxide nanorods on planar substrates and the modification of their field emission properties [51,52], it became possible to precisely control the morphology and size of MoO_2_ nanostructures, such as fabricating nanoparticles, nanorods, thin films, or other complex 2D/3D structures. The local electromagnetic field distribution can be optimized, and the SERS performance can be further enhanced. Through large-area chemical vapor deposition, Wu et al. [53] successfully fabricated a consistent slender molybdenum dioxide (MoO_2_) nanosheet layer. This substrate showcased remarkable signal strength uniformity throughout the detection zone, indicating superior performance homogeneity. The experiments have excellent signal homogeneity and exceptionally high sensitivity, with the lowest detectable concentration reaching 4 × 10^−8^ M (Figure 4a). In addition, the obtained enhancement factor is as high as 2.1 × 10^5^, and the substrate is even comparable to the SERS system using a noble metal film as the substrate. This enhancement is mainly attributed to the surface plasmon resonance effect by first-principles calculations and UV-Vis absorption spectroscopic characterization, which can be further enhanced by decreasing the thickness of the MoO_2_ nanosheets.

As mentioned, MoO_2_ is expected to perform well in practical detection applications based on optimized design and fine-tuning. Cao et al. [54] constructed vacancy-containing MoO_2−x_ (0 < x < 1) substrates by using MoO_2_ nanorods as the original nanostructures and generating the surface anoxia by the lithium metal grinding reduction (LMGR) process. Using R6G as a Raman probe, a greatly enhanced Raman signal with a low LOD of 10^−8^ M was achieved compared to the non-vacant MoO_2_ SERS substrate. This mechanism is based on the creation of deep energy level centers in artificial oxygen vacancies, which act as steps and provide an efficient pathway for electron transfer inside semiconductors, thus significantly enhancing the laser excitation conditions under the probability of electron jump inside the semiconductor under laser excitation conditions. Zhou et al. [55] plasmon Mo/MoO_2_ hybrid nanospheres were prepared using a simple hydrothermal self-assembly process. The Mo/MoO_2_ nanospheres assembled from plasmon Mo and MoO_2_ nanoparticles have a loose nanostructure exhibiting enhanced surface plasmon resonance (SPR) effect in the visible range, which exhibited the EM enhancement system, and were applied to molecular detection. The hybridized nanospheres showed a minimum LOD of up to 1.0 × 10^−10^ M for R6G and a maximum EF of up to 6.2 × 10^7^, and this was attributed to the synergistic plasmon effect between molybdenum and molybdenum dioxide (Figure 4b). Unlike the above, Liu et al. [56] homogeneous MoO_2_ nanospheres and ZnSe nanowire heterostructured composites were prepared hydrothermally. This MoO_2_/ZnSe nanocomposite donor-bridges-acceptor (D-B-A) system exhibited excellent SERS sensitivity and photocatalytic activity. The plasmon–nonmetallic/semiconductor nanomaterials synergistically enhanced, based on EM and CM, with a LOD of 8.79 × 10^−9^ M and a maximum EF of 5.03 × 10^5^ of MB.

Beyond molybdenum oxides, molybdenum sulfides and nitrides also display remarkable Raman enhancement capabilities. A single layer of molybdenum disulfide comprises three atomic strata (S-Mo-S) arranged in distinct sequences, forming three phases: 1T, 2H, and 3R. Chen et al. [57] prepared novel MoS_2_ nanosheets as a SERS substrate using a more straightforward and low-cost method. They proposed for the first time a new strategy for the large-scale synthesis of vertically aligned MS_2_ nanosheets with a 1T/2H hybrid phase (1T/2H-MS_2_), which can be directly grown on metal foils with vertically aligned structures by a one-step hydrothermal reaction (Figure 4c), with a detection limit of R6G as high as 5 × 10^−8^ M. Subsequently, Du et al. [58] prepared a new MS_2_ nanosheet as a SERS substrate with large-scale 1T/2H mixed phase (1T/2H-MS) using a quasi-metal-based microwave process to prepare MoN and Mo_2_C hollow spheres with large surface areas (108.7–125.6 m^2^∙g^−1^) and ultrafine nanoparticles (2–5 nm), which solved the limiting drawbacks of the high-temperature (>1000 °C), high-pressure (several GPa)) environment required for the synthesis of MoN and Mo_2_C [59], and provided an alternative route for gentle and rapid preparation of ultrafine nanocrystalline transition metal nitrides and carbides.

Remarkably, many researchers unveiled the substantial application prospects of molybdenum disulfide in the capacity of a SERS substrate. Fu et al. [60] recently reported a method for the preparation of molybdenum disulfide (MoS_2_) via a simple hydrothermal reaction, which can be easily exfoliated into monolayer nanosheets by ultrasonication even in the absence of any surfactant (MoS_2_NS_S_). The resulting MoS_2_NS_S_ contains two defects, one caused by doping high valence Mo atoms and the other by doping S_2_^2−^. The SERS activity notably escalated with the rise in high-valence Mo defects, achieved through meticulous adjustment of precursor Na_2_S and Na_2_MoO_4_ ratios.

In contrast, the SERS activity of MoS_2_ NS_S_ initially showed an increasing trend in the process of increasing the S_2_^2−^ defects but gradually decreased after reaching a certain threshold. This study reveals the effect of defects on the SERS activity of molybdenum disulfide. However, since many of the basal surfaces of MoS_2_ are catalytically inert, the limited number of active sites and low conductivity seriously hinder the practical application of MoS_2_ [61]. Therefore, exposing more catalytically active sites and modifying the surface electronic structure of MoS_2_-based catalysts are key to improving the performance of MoS_2_ photovoltaic electrodes. Achintya Singha et al. [62] reported an Au-MoS_2_ hybrid for SERS detection of bilirubin in serum down to 10^−7^ M. The Au-MoS hybrid was used to detect bilirubin in serum. These noble metals can generate LSPR, which promotes electron transfer in HER and improves the SERS sensitivity of MoS_2_. Arvind Kaushik et al. [63] synthesized molybdenum disulfide in different morphologies (flowers, interconnected nanoplates, and sheets) and used silver NPs to functionalize MoS_2_ nanoplates. MoS_2_-Ag nanocomposite SERS substrate successfully detected an ultra-low concentration of RhB dye at 10^−15^ M with an EF of 9.2 × 10^7^. However, optimizing molybdenum disulfide using noble metal doping also has limitations, such as economy, scarcity cost, and so on, so finding more cost-effective and sustainable optimization methods is necessary. Zhang et al. [64] designed a two-atom doping strategy (Figure 4d) and proposed a co-doping strategy for Ru and O-intercalated MoS_2_ (MSOR_x_) phase-free nanoparticles (where x = 0.5/1/1.5/2). This MSOR_x_ material utilizing a one-step hydrothermal method to accelerate charge transfer and establish more active sites exhibited good HER and SERS properties, with low overpotentials of 10 mA cm^−2^ for MSOR_1_ in acidic solution (197 mV) and alkaline solution (43 mV). The optimal MSOR_1_ has a high EF of 1.7 × 10^6^. The detection limit of bilirubin in serum is as low as 10^−10^ M, which can be used as a powerful substrate for the diagnosis of jaundice in clinical translations (Figure 4e). This novel bifunctional material achieved breakthroughs such as high-performance recyclability and improved durability. The SERS properties based on molybdenum metal are undoubtedly a great treasure, the development of which is still being explored.

### 2.4. W-Based Nanomaterials

**Figure 5:**
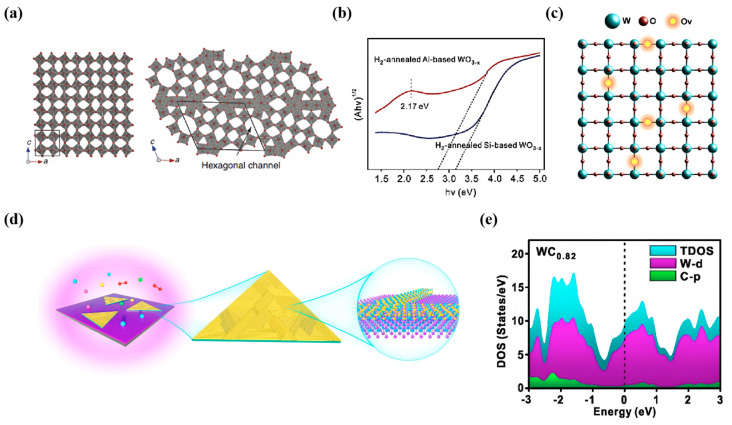
(**a**) Schematic structure of WO_3_ and W_18_O_49_ [66]. (**b**) UV-vis spectra of H_2_ annealed Al-based and Si-based WO_3−x_ films [67]. (**c**) Atomic modeling diagram of Vo randomly distributed WO_3−x_ [69]. (**d**) Schematic diagram of one-dimensional oriented WO_3−x_ nanowires synthesized from two-dimensional WSe_2_ flakes by oxygen plasma treatment [76]. (**e**) The electronic structure of WC_0.82_ was obtained by DFT simulation [77].

Due to its ample surface charge adjustability, morphological versatility, and molecular specificity, tungsten trioxide, an n-type semiconductor, found broad application in surface-enhanced Raman scattering. The enhancement of the Raman scattering effect of WO_3_ sputtering on a smooth substrate coated with a thin film of silver was first investigated by Thi [65], and the observed SERS effect appeared to be due to the formation of a tungsten bronze at the WO_3_/Ag interface. In 2015, Cong et al. [66] synthesized W_18_O_49_ with spiny, spherical, and sea urchin-like morphologies by a hydrothermal method. They used the vacancy-containing W_18_O_49_ as a substrate material to achieve a greatly enhanced SERS effect for the first time on a functionally enriched tungsten oxide material, with an EF of 3.4 × 10^5^ for R6G (Figure 5a). Under 532 nm laser light, the overall Raman enhancement in the W_18_O_49_-R6G system may be related to several charge transfer resonances. These include the exciton resonance associated with the W_18_O_49_ defect state (MEX), the molecular resonance of R6G, the photon-induced charge transfer resonance between matching energy levels of W_18_O_49_ and R6G molecules (mPICT), and the resonance attributed to ground-state charge transfer (mGSCT). Based on manipulating the intrinsic properties of semiconductors to synergistically enhance the PICT resonance process between the substrate and the analyte, Fan et al. [67] constructed a highly sensitive amorphous non-stoichiometric WO_3−x_ film as a SERS substrate. Oxygen-rich vacancies and amorphous-phase WO_3−x_ films were prepared using a highly reducing Al substrate and hydrogen annealing atmosphere. The fusion of the two properties, non-stoichiometric and amorphous, resulted in a tighter energy band spacing (2.75 eV), additional defect energy levels formed inside the energy bands, as well as stronger exciton resonance effects and a higher density of electronic states near the Fermi energy level. These unique properties construct effective charge escape channels and transfer pathways and strengthen the vibrational coupling mechanism, facilitating the photoinduced charge transfer process between the analyte and the substrate and its PICT resonance enhancement effect. The band tails were extended into the bandgap of the amorphous WO_3−x_, which resulted in the narrowing of the bandgap of the Al-based WO_3−x_ film by 0.40 eV compared to the Si-based one (Figure 5b). The detection limit of this WO_3−x_ film for R6G was as low as 10^−9^. The maximum EF was 1.1635 × 10^7^, which significantly improved the SERS effect. Subsequently, Liu et al. [68] prepared porous tungsten trioxide films by pulsed electrodeposition, which exhibited significant Raman enhancement (EF = 1.5 × 10^6^) for MB. They succeeded in firmly attaching the MB-labeled aptamer to the surface of the WO_3_ film through covalent bonding. They thus constructed an efficient aptamer sensor that exhibited excellent specificity and sensitivity for vascular endothelial growth factor (VEGF) with a detection limit as low as 8.7 pg/mL. Recently, Sun et al. [69] developed a simple solvothermal method for preparing high-performance SERS substrates with 3 nm width ultrafine WO_3−x_ nanorods. The lowest detection limit of R6G molecules on oxygen vacancy substrates can be as low as 10^−10^ mol/L, more than 10^2^ times higher than unmodified WO_3_. Oxygen vacancy (Vo) defects in semiconductors can create a surface state enrichment effect, which in turn enhances the density of states (DOS) inside the bandgap, in addition to the unsaturated nature unique to oxygen vacancies stemming from the presence of dangling bonds on their undercoordinated atoms. These properties endow the oxygen vacancies with the ability to adsorb efficiently and dynamically, enhancing the strength of the molecular bond between the adsorbate and the semiconductor surface (Figure 5c).

Meanwhile, the rapid development of tunable devices led to new advances in surface Raman enhancement of tungsten oxide. Zhou et al. [70] designed an electrically tunable Raman-enhanced substrate based on tungsten oxide (WO_3−x_). By controlling the leakage current of the oxide to program the defect density of the WO_3−x_ electrically, the SERS detection capability of the novel substrate can be instantly invoked, and the EF can be further precisely modulated. The initial EF of the substrate is 3.01 × 10^5^ when the leakage current is 1 × 10^−7^ A. As the leakage current level increases, the EF can be tuned to a higher level, reaching 1.14 × 10^6^ at a current of 1 × 10^−3^ A. In the same year, Hou et al. [71] reported a large-area hexagonal prune-like WO_3−x_ nanoarray prepared based on an aluminum nanobowl array substrate. They achieved a low detection limit of less than 10^−9^ M by tuning the tungsten magnetron sputtering time, and thus, the surface plasmon resonance localization to modulate the LSPR effect of the SERS substrate. In addition, they set up four groups of different enhancement effect modes. They explored the enhancement mechanism of the system in depth, revealing that there are interactions and influences between the LSPR effect and the photoconductive transient (PICT) phenomenon in the system. Using tungsten oxide as a research object, Cong et al. [72] developed an innovative strategy to ensure the semiconductor SERS substrate achieves the desired signal amplification, uniformity, and reproducibility performance. They combined a color-changing electrochromic mechanism with semiconductor SERS substrates by precisely controlling ions’ and electrons’ quantitative insertion and extraction processes, i.e., performing reversible reduction/oxidation operations to achieve substrate color changes. When the excitation wavelength of 532 nm is used, the tungsten oxide substrate, which was converted to a dark blue state after electrochromic treatment, shows a significant enhancement of the SERS signal intensity for several typical dye molecules, namely, R6G, CV, and Victoria blue B (VBB), which is much higher than that of the non-chromic-treated substrate. Systematically varying the negative voltage, they found a clear quantitative relationship between the SERS enhancement of colored substrates and the amount of inserted charge. By precisely regulating the amount of injected charge, the chemical structure and electronic states of semiconductor SERS substrates can be effectively tuned, which enables precise control and optimization of SERS activity. This achievement provides a new theoretical basis and technical path for designing and preparing high-performance and controllable semiconductor SERS substrates.

To solve the problem of detection strength of semiconductor substrates, semiconductor heterojunctions received attention. The composite heterostructures formed by WO_3_ and other metals exhibit excellent SERS activity, and the formed heterojunctions can effectively separate electron–hole pairs, reduce the electron–hole complexation, and improve the utilization of photogenerated electrons [73]. Shi et al. [74] used an electron-beam evaporation method to construct heterostructured WO_3_-TiO_2_ composite films. They varied the thickness of the TiO_2_ buffer layer and heat-treated the deposited samples. The introduced oxygen vacancy defects amplified the Raman scattering cross-section. The treated films showed a significant increase in the signal intensity for detecting methylene blue dye, which was enhanced by a factor of 2.7 compared to the untreated monolayer, attributed to the combined effect of oxygen vacancies and non-homogeneous structure. It also demonstrated good homogeneity and high reproducibility. Recently, Farha Naaz et al. [75] synthesized pristine and Ag-doped WO_3_ nanosheets using a hydrothermal method and deeply explored the multifunctional properties of these materials to achieve enhanced functionality in catalytic organic transformations and inefficient photocatalytic and electrocatalytic hydrogen precipitation reactions. The results show that the 1% silver-doped WO_3_ nanosheets possessed remarkable catalytic performance with 100% glycerol conversion and 90% triacetate selectivity. In the same year, Qian et al. [76] reported the synthesis of one-dimensional/two-dimensional WO_3−x_/WSe_2_ hybrid heterogeneous SERS materials with good controllability and reproducibility through oxygen plasma-treated mixed-dimensional WO_3−x_ nanowire/WSe_2_ heterostructures, where the one-dimensional WO_3−x_ nanowire pattern was arranged transversely along the triple symmetry direction of the two-dimensional WSe_2_ (Figure 5d). Even in mixed solution, the 1D/2D WO_3−x_/WSe_2_ heterostructure has a detection limit of up to 5 × 10^−18^ M for methylene blue molecules with an EF of 5.0 × 10^11^. In addition to tungsten oxides, Leilei et al. [77] reported a series of plasmon metal carbide SERS chips. They used a DC magnetron sputtering technique to deposit tungsten carbide on Si (100) wafers (Figure 5e), which has a carrier concentration of 4.32 × 10^23^ cm^−3^, sufficient to support LSPRs in the visible region. The EF of the WC_0.82_ chip is about 2.31 × 10^5^ at a concentration of R6G of 5 × 10^−8^ M, and it exhibits good signal uniformity and time stability. This technique applies to the large-scale fabrication of various carbide-based chips, encompassing tungsten carbide, niobium carbide, titanium carbide, and molybdenum carbide. Its scalability and versatility make it a preferred choice for industrial production of these advanced materials, which find critical applications in numerous sectors ranging from electronics and optics to catalysis and wear-resistant coatings.

### 2.5. Other Non-Noble Metal Nanomaterials

**Figure 6:**
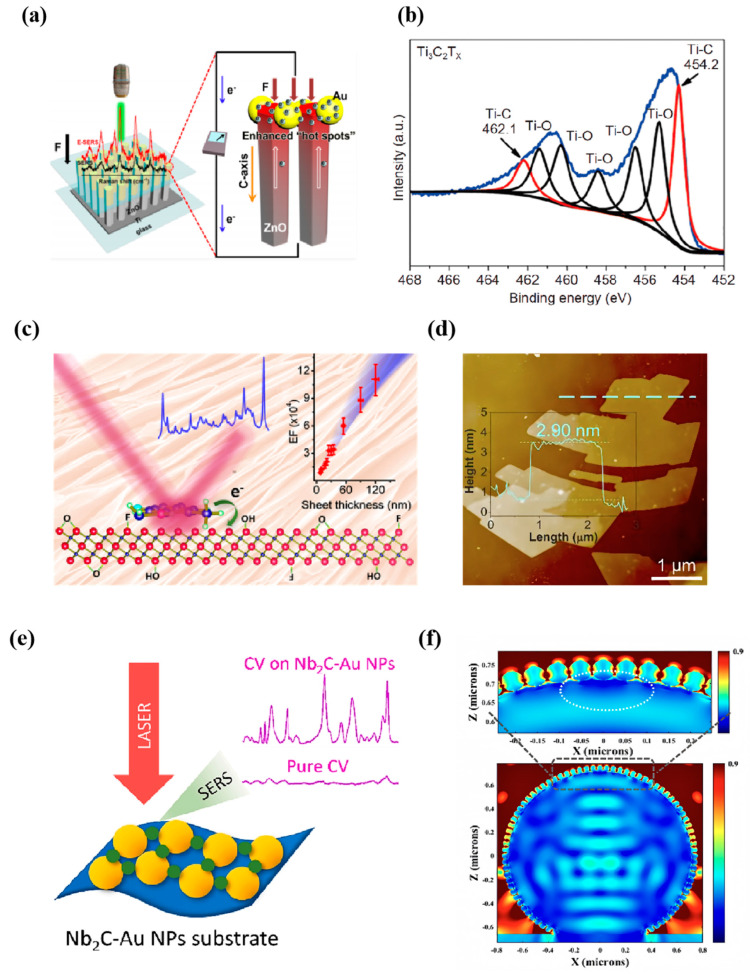
(**a**) E-SERS performance and composition of functional substrate layers [81]. (**b**) XPS spectra of monolayer Ti_3_C_2_T_x_ nanosheets under microwave heating [84]. (**c**) Schematic EF curves of the enhanced Raman mechanism of Ti_3_C_2_T_x_ nanosheets [85]. (**d**) AFM image of TiVC nanosheets [90]. (**e**) Schematic SERS enhancement of Nb_2_C-Au NPs [92]. (**f**) Simulated electric field distribution and local multiplicity on solid spheres of Ti_3_C_2_-Ag NPs [93].

More and more research is devoted to developing high-performance semiconductor-based SERS substrates. In addition to these more widely studied non-noble metal materials introduced above, there are some other non-noble metal substrates with good surface Raman enhancement effects, such as porous ZnO nanosheets [78], TiO_2_ nanostructures [79], Ta_2_O_5_ nanorods [80], NbS_2_, PbS, etc., among which the CM effect plays a dominant role in Raman enhancement. Xu et al. [81] designed and constructed an electrically modulated surface-enhanced Raman scattering (E-SERS) substrate, which is wearable and wireless, battery-free, and exhibits significantly improved detection sensitivity. They combined zinc oxide nanorods (ZnO NRs) with an asymmetric gold-decorated structure. They utilized controlled pressure generated by an external magnet to act on the substrate, thereby effectively modulating and enhancing the potential changes generated by the piezoelectric effect (Figure 6a). This design enables the E-SERS substrate to realize the dynamic modulation of SERS signals by changing the mechanical stress without needing a power supply, which provides new possibilities for portable sensing applications.

Thi Ha Tran et al. [82] proposed an effective strategy for preparing surface-enhanced Raman-scattering ZnO/Ag nanocomposites by combining the hydrothermal-assisted galvanic coupling effect and sputtering process. The optimal sputtering conditions to achieve the highest Raman enhancement were determined, and the optimal sputtering time was 70 s, which provided a strong methylene blue Raman signal. Recently, Wu et al. deposited a hydrophilic Ti_3_C_2_T_x_ film [83] on an array of superhydrophobic ZnO nanorods to create a novel SERS substrate with improved analyte affinity. It successfully overcame the previous difficulties in manipulating small-volume droplets on superhydrophobic substrates. The detection limit of this superhydrophobic substrate for R6G reached 10^−11^ M. In addition, the SERS activity in combination with ZnO/Ti_3_C_2_T_x_ suggests that it can also be used as an effective substrate for ultrasensitive environmental pollutant monitoring and bio-analyte detection.

Ye et al. [84] successfully developed a Ti_3_C_2_ monolayer nanosheet consisting of a nucleated two-dimensional electron gas structure (2DEG), a SERS substrate with excellent biocompatibility, low cost, and high sensitivity. They obtained Ti_3_C_2_ monolayer nanosheets with clean surface and high crystallinity by improving the chemical stripping technique and combining the microwave heating method (Figure 6b). The presence of nucleated 2DEG in this structure provides an ideal carrier transport path for the Ti_3_C_2_ nanosheets, which enables the highly crystallized monolayer Ti_3_C_2_ nanosheets to achieve significant Raman signal enhancement with an EF of up to 3.82 × 10^8^ for the probe molecule R6G, showing excellent SERS performance and stability. Limbu et al. [85] experimentally explored the SERS activity of titanium carbide (Ti_3_C_2_T_x_) nanosheets with varying thicknesses for the first time. The thickness of the nanosheets varied from 5 to 120 nm. The results show that the EF of the substrate to MB, the probe molecule, increased monotonically with the gradual increase in the thickness of the Ti_3_C_2_T_x_ nanosheets (Figure 6c). The Raman EF peaked when the thickness of the Ti_3_C_2_T_x_ film reached 2.0 um, which implies that the maximum Raman signal enhancement can be achieved by the Ti_3_C_2_T_x_ film with a thickness of 2.0 μm as a substrate. This thickness-dependent Raman enhancement phenomenon can be explained by the effective adsorption and intercalation behavior of MB molecules in the interlayer voids of Ti_3_C_2_T_x_. Further, by combining experimental observations with mathematical simulations, they identified the charge transfer mechanism as the main reason for the significant Raman enhancement effect on the surface of Ti_3_C_2_T_x_ material. Differently, Li et al. [86] noticed the variable valence properties of rare earth elements and doped rare earth element Ce into TiO_2_ using the sol-hydrothermal method to develop a high-performance semiconductor Ce_n_Ti_1−n_O_2_ nanoparticle substrate based on an energy level tuning strategy. The rare earth metal Ce can coexist in the semiconductor system in different valence states of Ce^4+^/Ce^3+^ to produce different doping energy levels. Various electronic arrangements, spawned by diverse energy levels, contribute to the intensification of interfacial charge transfer (CT) between the substrate and the molecular entities. The results indicate that Ce_0.010_Ti_0.990_O_2_ exhibits ultra-sensitive SERS activity with EFs as high as 2.2 × 10^6^, and the detectable concentration for MB probe molecules can be as low as 10^−10^ mol/L, which is comparable to that of some noble metal substrates.

MXenes, made of transition metal carbides and nitrides, recently became part of the SERS substrates alongside graphene and TMDs. Their formula M_n+1_X_n_T_x_ (n = 1–4) defines MXenes, where M is an early transition metal, X is C or N, and Tx represents surface terminations such as O, -OH, -F, and -Cl [87]. They showed great activity in surface Raman enhancement. Song et al. [88] achieved the controlled synthesis of two-dimensional ultrathin metallic niobium disulfide (NbS_2_) (<2.5 nm) with a large domain size (>160 μm). They investigated the SERS performance of this NbS_2_. They found that its detection limit was as low as 10^−14^ mol/L and that NbS_2_-based plasmon-free excitonic SERS substrates are used to differentiate between different types of red wines. Huang et al. [89] constructed mesoporous TiO_2_/Ti_3_C_2_ composite materials. Due to their material properties, these composites exhibit significantly enhanced light absorption capabilities and improved separation and transfer abilities of photocatalytic carriers. They achieved a photocatalytic degradation efficiency of MO at 99.6% within 40 min. Under visible light illumination (λ > 420 nm), the material demonstrated photocatalytic removal efficiencies of 99.2% for Rhodamine B (RhB) and 92.1% for tetracycline hydrochloride (TC), respectively. Additionally, He et al. [90] reported a one-step chemical etching method without ultrasonication and organic solvent insertion for large-scale synthesis of less-layered calcium titanate nanosheets with a high degree of crystallinity, deriving from the innovative ideas of Bimetallic MXene. The synthesized TiVC flexible film was used as a SERS substrate to detect R6G dye systematically. An ultra-high Raman enhancement of 3.27 × 10^12^ and femtomolar detection sensitivity was obtained (Figure 6d). This work provides a valuable guide for exploring other bimetallic and multimetallic solid solution MXenes with excellent SERS effects, such as TiNbC, VNbC, (Ti, V)_3_C_2_, and TiVNbMoC_3_.

Apart from this, Wu et al. [91] constructed a SERS proportional optosensor based on MXenes-loaded AuNP dimers with nanogaps. Through the hydrogen bonding and chelation interactions between the sh aptamer-modified AuNP dimer and MXenes nanosheets, the AuNP dimer/MXenes assembly with the highest Raman signal was developed to realize the trace detection of AFB1. In the same year, Yang et al. [92] prepared a fast, sensitive, and stable label-free nondestructive detection Nb_2_C-Au nanocomposite SERS platform by using the electrostatic self-assembly method (Figure 6e). The EM hot spots of Nb_2_C-Au NPs are significantly enlarged and swollen. At the same time, the surface Fermi energy level is reduced, and this synergistic effect can improve the system’s SERS performance. As a result, the detection limits for the dye molecules CV and MeB reached 10^−10^ M and 10^−9^ M, respectively. This work may expand the application of MXene-based materials in the field of SERS. Subsequently, Moru Yang et al. [93] proposed a strategy for preparing three-dimensional MXene hollow spheres encapsulating silver nanoparticles (Ti_3_C_2_-AgNP HSs) using a sacrificial template method (Figure 6f). Compared with the Ti_3_C_2_ solid structure, the 3D Ti_3_C_2_ HSs have more exposed active sites and better light trapping ability due to hollow spheres’ good light trapping ability and the strong LSPR effect of AgNP. It is shown that the hollow structure of Ti_3_C_2_-AgNP HSs has superior sensitivity and lower detection limit, which makes up for the previous limiting studies of MXenes materials based on blocks and nanosheets and develops a new direction to improve the Raman performance of MXenes surfaces. This study has a promising application in the in situ detection of catalytic reactions.

## 3. Applications

Trace detection is essential in medical diagnosis, food safety, environmental science, and coronavirus detection [94,95,96]. Employing surface-enhanced Raman spectroscopy for detecting various substances offers a universal, efficient approach to discerning molecular fingerprint information, both label-free and labeled SERS methods were applied to the biomedical field. It is thus widely used in detecting trace substances [97]. Non-noble metal materials often have unique chemical properties on the surface that differ from those of noble metals. These can provide more active sites for modification and functionalization and are conducive to designing diversified probes and recognition systems to improve selectivity and sensitivity. In addition, certain non-noble metal materials have excellent mechanical strength and flexibility, which makes them suitable for fabricating flexible electronic devices or portable sensors for rapid on-site detection and applications in complex environments. In particular, the good biocompatibility of non-noble metal matrices led to significant breakthroughs in bioimaging, cancer diagnosis, and treatment [98,99,100]. At the same time, the unique interfacial interactions between semiconductor materials and targets contribute to important selective detection [5]. Here, we will detail the application of non-noble metal materials as SERS substrates for detecting trace assays of different biomedical substances in recent years.

### 3.1. Biomarkers Detection

Cancer is one of the most severe health challenges worldwide, posing a significant threat to human life and well-being [101,102]. The timely identification of cancerous cells or tumors before symptoms manifest can lead to less invasive treatments, higher cure probabilities, and a better quality of life for patients. SERS has broad application in various bio-detection strategies, including molecular visualization, tumor characterization, medication surveillance, cell profiling, exosome detection, and other biomedical studies. SERS technology can detect characteristic fingerprint peaks of different types of cells, proteins, miRNAs, and DNA [103], so it has unique advantages in biomarker detection [104]. The non-noble metal SERS substrates that emerged in recent years gradually became popular nanomaterials in the field of SERS due to their obvious tunability and unique biomolecule compatibility. Non-noble metals SERS substrates have broad application prospects in the SERS and materials fields [9,105].

#### 3.1.1. CTC

**Figure 7:**
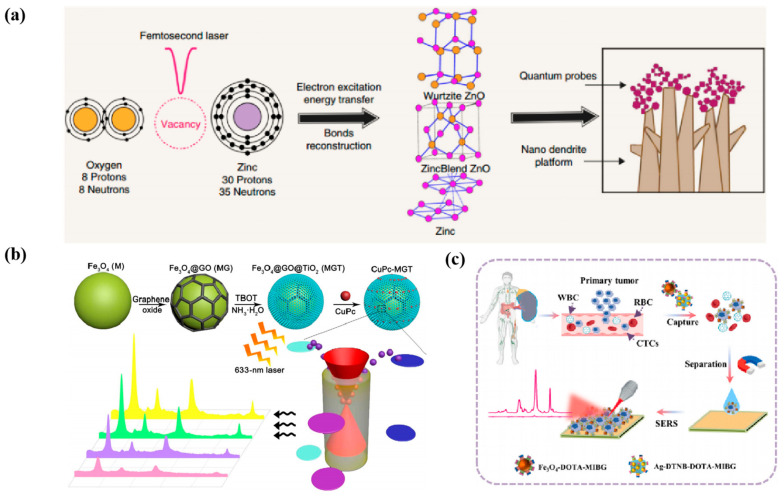
(**a**) Formation of ZnO-based semiconductor quantum probes for SERS and nano dendrimer platforms for cell adhesion using femtosecond laser interaction [108]. (**b**) Schematic of the MGT substrate’s synthesis process and enhancement mechanism [109]. (**c**) Mechanism of action of dual-targeted SERS cell sensors [111].

Circulating tumor cells (CTC) are cells shed from the primary tumor, carrying the biological characteristics of the primary tumor and circulating throughout the body with the blood. These cells are indicators of the unique characteristics and behavior of the tumor and have a significant role in cancer prevention, detection, and treatment [106]. Wu et al. [107] developed three different shapes of novel gold nanoparticles (spherical, rod, and star) for the detection of CTC in the blood, and the AuNS-MBA-rBSA-FA particles became the best, with a detection limit as low as 1 cell/mL. Haldavnekar et al. [108] developed a label-free, biocompatible ZnO-based 3D semiconductor quantum probe for label-free in vitro diagnosis of cancer. Defects on the surface of the quantum-sized 3D ZnO SERS probe, such as oxygen deficiency and layer mismatches, lead to exponential enhancement of the SERS signal. This allowed differentiation between cancerous and benign states by analyzing the lipid-to-protein peak intensity ratio (I_1445_/I_1654_). The quantum probe decorated on a nano dendritic platform (Figure 7a) essentially mimics the extracellular matrix, allowing self-targeting, cell adhesion, and proliferation, and this label-free multiplexed SERS assay is applied to the in vitro cancer diagnosis. Additionally, Feng et al. [109] innovatively synthesized a novel ternary heterostructure: a Fe_3_O_4_@GO@TiO_2_ substrate. The MGT enhancement factor of this SERS substrate was as high as 8.08 × 10^6^ due to the resonance effect of CuPc, CT between GO and TiO_2_, and enrichment from porous TiO_2_ shell layers (Figure 7b). Following this, they developed a novel plasmon-free SERS probe based on nanocomposites for sensitive and selective detection of various types of TNBCs at the single-cell level and for the assessment of the expression of programmed cell death receptor ligand 1 (PD-L1) with a detection limit as low as ~3 cells. MGT nanocomposites exhibit excellent recoverability under visible light, which is clinically valuable for diagnosing and treating TNBC in humans and can be extended to detecting other microscopic cancer biomarkers to diagnose cancers and other diseases reliably. Xu et al. [110] utilized a microfilter on a B-TiO_2_ bioprobe to isolate and directly detect CTC in peripheral blood in situ at single-cell resolution and to separate CTC from blood microfilters based on CTC size and deformation differences. Experiments demonstrated that the B-TiO_2_-AR-PEG-FA SERS bioprobe distinguishes between folate receptor-positive CTC and peripheral blood cells, excluding false-positive interference from leukocytes with high reliability. Pheochromocytoma (PCC) is a rare tumor. Meng et al. [111] constructed a dual-targeted SERS cellular sensor, which can achieve susceptible detection of pcc-CTC in blood samples. Two target-modified magnetic probes based on a Fe_3_O_4_-DOTA-MIBG and Ag-DTNB-DOTA-MIBG and signal amplification probe acted synergistically for the magnetic separation and SERS analysis of pcc-CTC in peripheral blood, respectively (Figure 7c). Ag nanocubes (AGNC), as a Raman-active substrate, were able to provide an enhanced electromagnetic field, and showed good linearity in the range of PC12 samples with concentrations of 3.0~3.0 × 10^6^ cells·mL^−1^, with a detection limit of 1 cell·mL^−1^.

#### 3.1.2. Exosome

**Figure 8:**
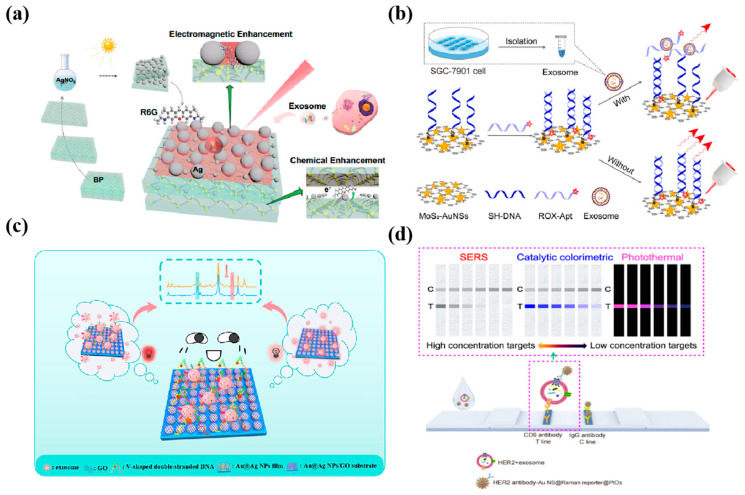
(**a**) Schematic diagram of the synthesis and application of Ag/BP-NS SERS sensor [99]. (**b**) Fabrication of MoS_2_-based aptasensor for exosome detection [113]. (**c**) Schematic diagram of the Au@Ag NPs/GO) biosensor [114]. (**d**) Principle of the Au@Raman Reporter@PtOs-driven LFA for catalytic colorimetric, SERS, and photothermal signal detection of breast cancer exosomes [115].

Exosomes are small vesicles secreted by cells. The detection of exosomes has the advantages of lower cost and minimal invasiveness in disease diagnosis, treatment, and monitoring [112]. Lin et al. [99] constructed a “multilayer nutcake” nanostructure SERS sensor by embedding silver nanoparticles into multilayered black phosphorus nanosheets (Ag/BP-NS) via light-driven chemical reduction (Figure 8a), the lowest detection limit of this substrate for R6G was 10^−20^ mol/L. With machine learning, Ag/BP-NS has good biocompatibility and homogeneity in practical applications. It can detect individual tumor exosomes by SER imaging to distinguish and identify tumor exosomes of different cell lines based on a support vector machine of Ag/BP-NS substrate. Pan et al. [113] constructed a novel SERS nanoprobe by modifying AuNSs on MoS_2_ nanosheets. They assembled ROX (6-carboxy_−x_-rhodamine)-labeled aptamers (ROX-Apt) on the surface of MoS_2_-AuNSs (Figure 8b), which were used to recognize the specifically bound transmembrane protein CD63 (a representative surface marker of exosomes). ROX-Apt forms a double chain with its sulfhydrylated complementary sequence, which is immobilized on the basal surface of MoS_2_-AuNSs, and the incorporation of exosomes results in the formation of the ROX-Apt-exosome complexes. The addition of exosomes leads to the formation of ROX-Apt-exosome complexes and the detachment of ROX-Apt from the surface, forming a “signaling shutdown” mechanism, which provides the sensor with high sensitivity and selectivity for detecting gastric cancer-associated exosomes. Zhang et al. [114] combined the electromagnetic enhancement of SERS by Au@Ag nanoparticles (Au@Ag NPs) and the high specific surface area of graphene oxide for DNA adsorption to construct a proportional SERS biosensor (Figure 8c). Briefly, they chose two breast cancer-associated proteins, EpCAM and HER2, as recognition sites for exosome detection, and formed v-double-stranded DNA with the aptamer, which triggered the competitive response of exosomes. The strategy has a broad linear detection range (2.7 × 10^2^~2.7 × 10^8^ particles/mL) and a lower detection limit as low as 1.5 × 10^2^ particles/mL without any nucleic acid amplification. This method achieved the first dual aptamer recognition of breast cancer-derived exosomes, which helps to distinguish pancreatic cancer patients from healthy individuals accurately. Then, Lin et al. [115] developed a new composite Au nanostar@PtOs nanocluster by growing PtOs bimetallic nanoclusters around Au nanostars (Au NS) for multi-mode lateral flow analysis (LFA) detection. The bimetallic nanocluster doping strategy slowed the SERS decay, and the simulated pod catalytic activity significantly improved compared to the monometallic [116,117]. Its detection limit in colorimetric/SERS/temperature mode was 2.6 × 10^3^/4.1 × 10^1^/4.6 × 10^2^ exosomes/μL, respectively (Figure 8d), which was much higher than that of ordinary gold nanoparticles LFA (~10^5^ exosomes/μL). Meanwhile, this strategy’s fingerprinting molecular recognition capability can directly distinguish exosome phenotypes from different breast cancer cell lines.

#### 3.1.3. miRNAs

MicroRNAs (miRNAs) are a class of endogenous, highly conserved small, non-coding, single-stranded RNA molecules. From the embryo’s formation to the individual’s maturation to the onset of aging and disease, miRNAs regulate cells through gene expression, which is vital in biological development [118,119]. The mainstay of surface Raman scattering for miRNA detection is still noble metal materials [120], and other non-noble metal types of nanomaterials usually rely on CM rather than EM. Ma et al. [121] utilized the combination of the electronic effect of GO and the phonon structure of gold nanoparticles to develop a pyramidal probe of gold upconverted nanoparticles, which can detect two types of targets. Due to good biocompatibility, this GO-Au NP structure allows in situ quantitative detection of miR-21 in live target cells, where the unique electronic synergy of GO and gold nanoparticles produces strong activities.

Similarly, Liu et al. [122] developed a co-calibrated SERS strategy based on the ternary system of MXene/MoS_2_@Au nanoparticles (MMA). Firstly, the average intensities of the three characteristic Raman peaks (382 cm^−1^ and 402 cm^−1^ of MoS_2_ and 611 cm^−1^ for MXene) are chosen as the benchmark, avoiding the peak intensities’ uncertainty. Secondly, the average gap between the “hotspots” embedded on the MXene/MoS_2_ surface is 2.2 nm, uniform, dense, and highly numerous, thus achieving an ultra-low detection limit of 6.61 am for miRNA-182. An in situ platform is reported to extract exosomal miRNAs from serum samples [123]. First, synthetic locked nucleic acid (LNA)-modified Au@DTNB induced hotspot SERS signals. Then, Fe_3_O_4_@TiO_2_ nanoparticles were added to enrich exosomes further. The detection limit was 0.21 fM, better than or comparable to previously reported in situ methods such as qRT-PCR. Experiments using exosomal miRNA-10b identified pancreatic ductal adenocarcinoma (PDAC) patients with 99.6% accuracy. This non-invasive liquid biopsy method is suitable for clinical cancer diagnosis. In addition, Jiang et al. [124] developed 3D WO_3_ hollow microspheres as SERS-active substrates for detecting miRNA 155. By using 3D hollow microspheres with small band gaps and abundant surface defects, the fluorescence resonance energy transfer (CT) effect could be increased. Meanwhile, they constructed a SERS biosensor based on 3D WO_3_ hollow microspheres using a catalytic hairpin assembly (CHA) strategy for the highly sensitive detection of miRNA 155 with a lower detection limit of 0.18 fM, which is better than the previous report.

It is very noteworthy that microRNA discoverers were awarded the 2024 Nobel Prize in Physiology or Medicine. Victor Ambros and Gary Ruvkun’s amazing discovery revealed a whole new dimension of gene regulation, and miRNAs proved to be fundamentally important for the development and functioning of organisms [125,126]. Based on this, we think that detecting miRNA molecules with SERS can obtain even more heat in the future.

#### 3.1.4. Protein Tumor Markers

**Figure 9:**
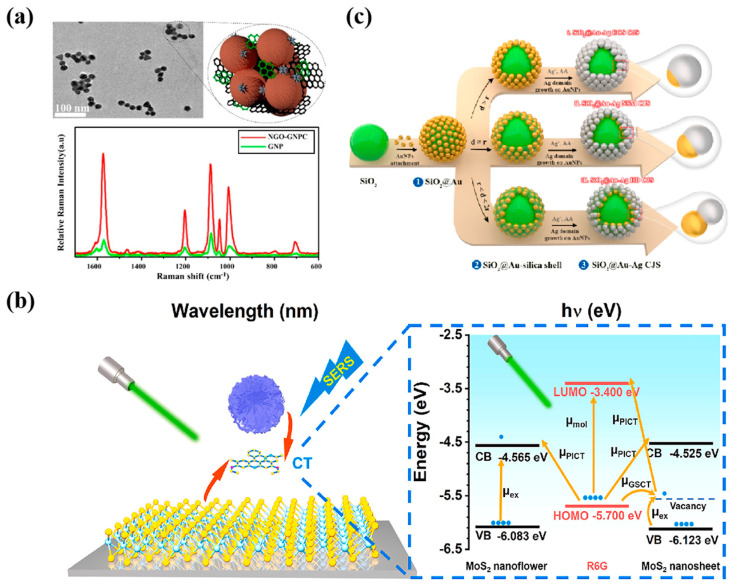
(**a**) TEM images and Raman spectra of NGO-GNPCs [128]. (**b**) Schematic energy band diagram of the charge transfer process in the ternary system of R6G, MoS_2_ nanoflowers, and nanosheets [129]. (**c**) Schematic illustration of significant steps involved in the synthesis of SiO_2_@Au-Ag Janus CJS [130].

Vascular endothelial growth factor (VEGF) is a key angiogenic marker that promotes vascular endothelial cells’ growth, survival, and proliferation, facilitating new blood vessel formation [127]. In glioblastoma (GBM), VEGF acts as an essential signaling protein. Liu et al. [68] prepared a porous WO_3_ thin-film SERS sensor modified with MB by pulsed electrodeposition, which can be used for the SERS detection of VEGF, a glioblastoma biomarker, in human serum, in which MB serves as the adsorbed molecule of the substrate. The sensor was highly selective for the detection of VEGF, with a detection limit down to 8.7 pg·mL^−1^. Ali et al. [128] reported clusters of gold nanoparticles (GNPs) induced by graphene oxide at 120 nm. NGO-GNPCs nanoprobes showed a 3- to 4-fold increase in the SERS signal compared to a single GNP (Figure 9a). They were used together with magnetic beads (MBs) to detect immunoglobulin G (IgG). ngo-GNPCs, IgG, and MBs formed a sandwich-type immune complex, and the Raman intensity was linearly correlated with the concentration of IgG. The detection limit was as low as 0.6 pM. This is the first time researchers used ngo to induce a cluster of GNPs with hydrophobic groups through non-covalent interactions. Furthermore, the Raman intensity was linearly correlated with the IgG concentration, with a detection limit as low as 0.6 pM. This is the first time researchers induced clusters of GNPs with hydrophobic groups by ngo through non-covalent interactions.

Similarly, Jiang et al. [129] hydrothermally synthesized sandwich immune complexes consisting of MoS_2_ nanoflowers and nanosheets, which could sensitively and precisely monitor carbohydrate antigen 19-9 (CA19-9). Photon-induced CT (μ_PICT_) and ground-state CT (μ_GSCT_) under 532 nm excitation can synergistically promote the SERS effect (Figure 9b), and combined with the strong molecular enrichment on the surface of the two-dimensional MoS_2_ material, the sensor achieves an EF value of up to 10^5^. It was demonstrated that the MoS_2_ sandwich sensor could estimate the concentration of the target CA19-9 in human serum samples more sensitively in the clinic than the conventional chemiluminescence immunoassay (CLIA) and showed better biocompatibility in practical use. Nearest, Hao et al. [130] developed an exclusive core-Janus satellite (CJS) assembly using a layered assembly strategy (Figure 9c). Au-Ag Janus satellites were used to vertically self-align on the core surface, and combined with a SiO_2_ asymmetric mask to form a series of Janus structures with different morphologies. The unique heterojunction morphology allows the SiO_2_@Au-Ag CJS assembly to have two types of plasmon nanogaps, including internal gaps in individual Janus and internal nanogaps between neighboring Janus, increasing the ‘hotspot’ with an EF as high as 3.8 × 10^8^. The lowest detection limit for CA19-9, a pancreatic cancer marker, was 3.67 × 10^−5^ IU·mL^−1^, and the detection range was 3 × 10^−5^~1 × 10^4^ IU·mL^−1^, which were at the forefront of relevant reports.

#### 3.1.5. Other Biomarkers

**Figure 10:**
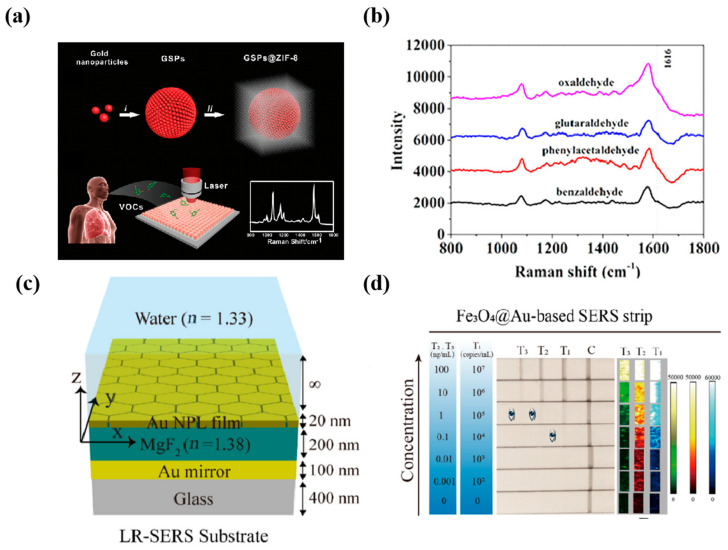
(**a**) Schematic diagram of synthesized GSP@ZIF-8 core–shell structure and SERS detection [131]. (**b**) Raman spectra of the glyoxal, glutaraldehyde, benzaldehyde, and phenylacetaldehyde at the concentrations of 1.0 ppb [133]. (**c**) Schematic of the structure of the LR-SERS substrate [136]. (**d**) Photographs of Fe_3_O_4_@Au-based SERS strips and corresponding SERS mapping images of three T lines for different concentrations of H1N1, SARS-CoV-2, and RSV [137].

To fulfill the need for better biocompatibility with biological samples as well as spectral stability and reproducibility, non-noble metal substrates were gradually applied to the detection of various disease markers. Their Raman enhancement effect proved effective in detecting other biomarkers, such as volatile organic compounds (VOCs). People who smoke tend to have higher levels of aldehydes in their exhaled breath than ordinary people. These aldehydes are by-products of lung cancer cell metabolism in a way. Qiao et al. [131] designed a specific core–shell three-dimensional structure to detect VOCs gaseous aldehydes (Figure 10a), in which ordered gold superparticles (GSPs) provided SERS hotspots to enhance the surface Raman scattering, and metal–organic frameworks (MOFs) were used to inhibit attenuation of the electromagnetic field around the surface of the GSPs, which made the gas molecules flow slow down when passing through, and analytes more fully adsorbed onto the SERS substrate. Aldehydes gaseous compounds were selectively trapped onto specific substrates by reacting with the Schiff base of the Raman active probe molecule p-aminothiophene (4-ATP). This technique has great potential for early lung cancer screening. Additionally, Su et al. [132] hydrothermally synthesized metal-free, Ni-doped MoS_2_ nanoflowers (NFs) and optimized them by adjusting the doping concentration, which resulted in an EF as high as 3.56 × 10^5^. The catalytic performance of the doped MoS_2_ NFs for MB was improved by 46.73% compared with that of pure MoS_2_ NFs, which promotes the application of MoS_2_ NFs in biosensors. Specific recognition is essential for the detection of individual substances, Wen et al. [133] synthesized a renewable hierarchical porous CuFeSe_2_/Au heterostructured nanosphere to detect aldehydes and lung cancer cells. Due to the presence of many cavity traps on the surface of the nanospheres, gaseous aldehydes pass through the surface of the nanospheres and undergo a ‘cavity vortex effect’, which leads to a longer gas reaction on the surface, with a detection limit of 1 ppb (Figure 10b). This innovation is the first time that cancer markers are detected with a reproducible nanoprobe. Similarly, Dharmalingam et al. [134] proposed the concept of atomic defect enhancement for quantum probe Raman scattering (DERS) at the molecular level, which can be used for label-free Raman detection of biomarkers such as ATP and EGFR peptides with low Raman cross sections. In addition, the researchers used graphene as a substrate to prepare SERS substrates for label-free detection of hemoglobin (Hb) and albumin (Alb) in blood. This is also the first example of demonstrating the enhancement of Raman signals when biomolecules are in contact with graphene [135].

The coronavirus disease (COVID-19), starting in 2019, poses a severe challenge worldwide. Currently, reverse transcription polymerase chain reaction (RT-PCR) is the most widely used standard for diagnosing the disease. However, RT-PCR is costly, complex, and technically demanding, so developing new viral assays is urgent. Wu et al. [136] developed a novel 2D thin film substrate using self-assembly. The long-range SERS (LR-SERS) substrate has an Au nanoplatelet film/MgF_2_/Au mirror/glass structure to enhance remote SERS generated by an extended electric field (Figure 10c). This fast and accurate reagent-free SERS assay for S proteins achieves a low detection limit of 9.8 × 10^−11^ g/mL in practical applications. In addition, Liu et al. [137] presented a multichannel SERS-LFA test strip based on Fe_3_O_4_@Au MNPs for the simultaneous ultra-sensitive detection of three respiratory viruses (H1N1/SARS-CoV-2/RSV). The immunomagnetic nanomaterial Fe_3_O_4_ NPs can capture and enrich the target analytes directly in the sample without pretreatment. After several repeated experiments, this SERS test paper showed high monodispersity and strong enhancement ability, and the detection limit and sensitivity were significantly improved (Figure 10d). The method can be applied to rapid virus detection under field conditions. Table 1 compares recent nanomaterials and properties used for SARS-CoV-2 detection. As shown in Table 1, it is still the noble metal substrates that are more dominant in SARS-CoV-2 detection, but more and more hybrid systems with non-noble metal/noble metal substrates are performing well in SERS detection of biomarkers. This implies that non-noble metals are gradually occupying an important position in virus detection.

### 3.2. Contaminant Detection

Yearly rising emissions of volatile organic compounds (VOCs), pollution levels of heavy metal ions, and antibiotic residues are increasing pressure on ecosystem cycles and public health. In addition, pesticides, illegal additives, foodborne pathogens, antibiotics, and other contaminants in food greatly threaten human health [144]. Therefore, the challenge of detecting these pollutants efficiently and rapidly remains to be addressed. As a versatile analytical technique, SERS is regarded as an ultra-sensitive detection tool for the biological fingerprints of many molecules compared to traditional detection methods (voltammetry, chromatography, capillary electrophoresis, etc.) [145,146,147]. Non-noble metal nanomaterials, which are stable, non-toxic, non-energy-consuming, environmentally friendly, and inexpensive, showed a broad application potential in pollutant identification and detection. Some non-noble metal SERS substrates possess various morphologies and structures, such as nanoparticles, nanowires, and films. These morphologies are advantageous as they increase the specific surface area, offering a more practical area for detection.

#### 3.2.1. Volatile Organic Compounds

**Figure 11:**
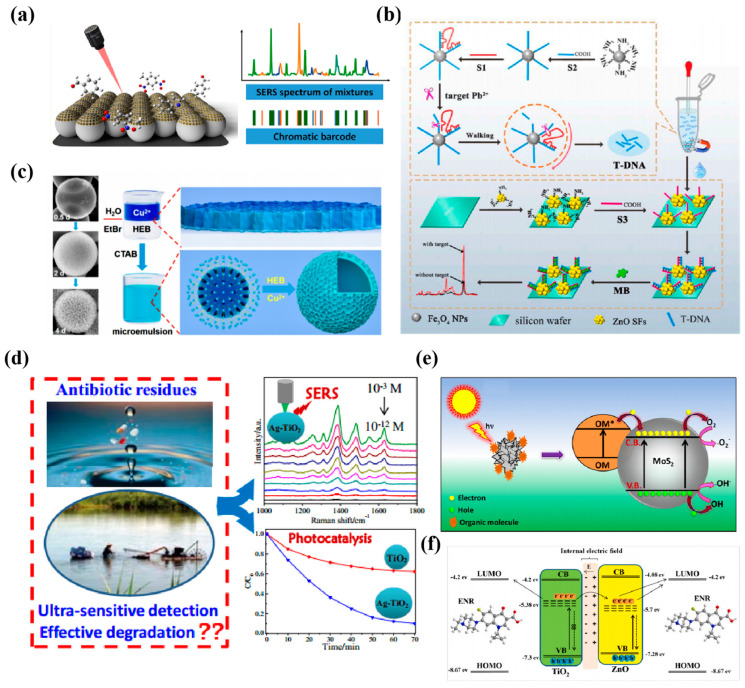
(**a**) Schematic diagram for detecting mixtures of multiple VOCs [152]. (**b**) Schematic diagram of the SERS biosensor for Pb^2+^ detection [156]. (**c**) Schematic diagram of the formation mechanism of GDY HSs [28]. (**d**) Schematic diagram of Ag-TiO_2_ nanosubstrate for SERS detection [161]. (**e**) Schematic of charge transfer mechanism between organic pollutants and MoS_2_ nanoflower photocatalysts under sunlight [162]. (**f**) Schematic of SERS mechanism of ENR on TiO_2_/ZnO heterojunction [165].

Song et al. [148] prepared heterostructure SnO_2_/MO_x_ (i.e., M = Zn, Ga, and W) nanotubes (NTs) and nanofibers (NFs) and constructed gas sensor arrays by one-step electrostatic spinning followed by calcination synthesis, which enabled effective and selective detection of hazardous gases, such as ethanol, acetone, and xylene, with a range as low as 10 ppm. Combining multiphase photocatalysis (photocatalytic degradation) and surface-enhanced Raman spectroscopy (dye detection), István Székely et al. [149] optimized the heterogeneous structure of Au/TiO_2_/WO_3_, and under UV irradiation, the composite of Au/TiO_2_/WO_3_ showed 96.6% removal of OA and 99.0% removal of phenol (PHE), and it also produced an intensity of the Raman signals of pollutant molecules. The detection limit for crystalline violet was 10^−8^ M. Ma et al. [150] prepared WO_3_ nanosheets by hydrothermal method, ball-milled and dispersed into deionized water, and deposited 36 layers of gas-sensitive films on Al_2_O_3_ ceramic sheets with Pt electrodes, encapsulated as a gas microsensor, and performed modular and unmanned tests on a high-throughput screening platform for four typical VOC gases. The results show that WO_3_ nanoplates containing 0.3 mol% Pd showed the highest response (R_a_/R_g_ = 131.2) to 100 ppm xylene at 250 °C, in which the ratio of the resistance of the sensor in air (R_a_) to the resistance in the target gas (R_g_) is defined as the response value of the sensor (S). Sugahara et al. [151] mixed graphene semiconductor and three-dimensional (3D) metal-oxide MoO_x_ nanorods networks to design a hybrid gas sensor with switching charge carriers. A significant feature of this sensor is that based on the semiconducting as well as the field defect doping nature of graphene, the hybrid h-Gr/MoO_x_ device exhibits back-and-forth between n-type (electrons) and p-type (holes) conductivity in response to electric field effects. In addition, with the fine nanostructure of MoO_x_ nanorods, the device has an ultra-large specific surface area favorable for the adsorption of gas molecules and a fast response rate with a low impedance of 10^−2^ Ω and a high-speed response/recovery time of about 20–30 s at a gas concentration of 400 ppm EtOH. Yang et al. [152] constructed a colloidal self-assembly-prepared honeycomb 3D substrate, bonded ultra-flexible Ti_3_C_2_T_x_ MXene onto the surface of the 3D honeycomb arrays, and introduced it into a microfluidic chip to prepare a SERS-Vortexene chip (Figure 11a). This 3D substrate with ordering can effectively provide a strong and uniform SERS effect and can spontaneously form an in situ vortex flow field in the nanostructured SERS hotspot region. This property is conducive to significantly enhancing the residence time of molecules on it. The flexible Ti_3_C_2_T_x_ MXene chip preserves the complex nano-microstructure in the 3D substrate. It maintains the vortex flow field with a low LOD of 10–50 ppb for three typical VOCs, namely, 2,4-dinitrotoluene (DNT), aromatic benzaldehyde, and indole.

#### 3.2.2. Heavy Metal Contaminants

Lead, arsenic, mercury, and other heavy metals are some of the most deleterious environmental pollutants and potent human carcinogens. The ubiquitous presence of these heavy metals in soils, water bodies, and the atmosphere necessitates stringent regulatory actions to alleviate the harm they inflict upon human health and ecological systems [153,154]. The application of the SERS technique to monitor heavy metal pollutants became increasingly widespread. For example, Parveen et al. [155] prepared ZnO@SWCNTs nanocomposite membranes with ZnO NPs functionalized with single-walled carbon nanotubes (SWCNTs) using thermal evaporation technique for the detection of various heavy metal ions in aqueous media. The sensor showed high selectivity and 0.225 nM detection limit for Pb^2+^ ions. Similarly, Zhang et al. [156] constructed a novel SERS biosensor for sensitive detection of Pb^2+^ using calcined ZnO semiconductor submicron flowers (ZnO SFs) as the substrate (Figure 11b). The calcined ZnO SFs have a smaller bandgap, which promotes the coupled resonance in the molecule–semiconductor system to generate highly efficient CT leaps and improve the biosensor’s sensitivity. Interestingly, a free-legged DNA walker was used to convert trace Pb^2+^ into trigger DNA (t-DNA) to achieve a sensitive detection of Pb^2+^ as low as 3.55 pM. Zhang et al. [28] synthesized for the first time highly crystalline GDY hollow spheres (HSs) with an ultrahigh-specific surface area (1246 m^2^/g). GDY HSs have an ultrahigh-specific surface area (Figure 11c), with potential water purification and Raman-sensing applications. The large-area graphene substrate has both physical and chemical adsorption. They prepared 20 mL of aqueous solution of organic dyes with health hazard concentration and dispersed GDY HSs (50 mg) uniformly in the hazardous sample. The GDY HSs was removed by centrifugation after stirring for 10 min, and the resultant experiments show that its ion removal efficiency for heavy metals in water, such as Hg^2+^, Pb^2+^, AsO_4_^3+^, CrO_4_^2+^, Cr^3+^, Cd^2+^, Ni^2+^, and Co^2+^ ranged from 92% to 99%. As an example, Ji et al. [157] demonstrated that semiconductor-enhanced Raman spectroscopy can determine Cr (VI) in water by using the charge transfer complex alizarin red S (ARS)-sensitized colloidal TiO_2_ NPs. Samriti et al. [158] synthesized TiO_2_ NRs with a band gap of 2.95 eV by acid immersion treatment with hydrothermal method and doped by 1–5 mol% of Ta. It was found that 5% Ta-doped TiO_2_ (5TaTiO_2_ NRs) exhibited better optical properties and SERS activity for detecting MB molecules in an aqueous solution. The doped TiO_2_ NRs showed better optical performance and SERS activity for MB dye molecule photodegradation. These doped TiO_2_ NRs showed excellent photocatalytic activity and long-term stability.

#### 3.2.3. Antibiotic

The misuse and overconsumption of antibiotics soared in recent years, posing significant risks to health and the environment. This practice fosters antibiotic-resistant pathogens, complicates medical treatments, and pollutes drinking water supplies [159,160]. Therefore, research focused on ensuring the rational use and effective monitoring of antibiotics. Recent studies showed that non-noble metal SERS substrates can effectively detect antibiotics. Wang et al. [161] prepared Ag-TiO_2_ (Ag synchronously deposited and doped with TiO_2_) nanoparticles SERS-active substrate. This Ag-TiO_2_ nanoparticles realized diflufloxacin hydrochloride, ciprofloxacin, enrofloxacin, danofloxacin, and enrofloxacin (five residual antibiotics) in actual water samples with susceptible SERS detection with detection limits as low as 10^−11^~10^−12^ orders of magnitude (Figure 11d). This method can accurately distinguish each antibiotic from mixed antibiotic residue samples, which is of great significance for environmental detection and ecological protection. Singh et al. [162] prepared molybdenum disulfide consisting of MoS_2_ flakes with tunable surface areas (5–20 m^2^/g) using hydrothermal method nanoflowers, which realized ultrafast SERS detection of various antibiotics and the dye adsorption capacity was positively correlated with its surface area. The MoS_2_ nanoflowers showed good photodegradation performance for fixed concentration solutions of MB, R6G, MO, and OTC-HCl molecules, which could be completely degraded within 12 min, 45 min, 30 min, and 60 min, respectively. Molybdenum disulfide nanoflowers with high surface area adsorb pollutant molecules more efficiently by providing available active sites within the sample, migration of pollutants occurs from the HOMO to LUMO level, and the electrons are transferred from LUMO to the conduction band of MoS_2_, increasing the electron density (Figure 11e). Arun Kumar et al. [163] studied the SERS performance of anisotropic silver nanoprisms (Ag NPrs) attached to the Cu_2_O microsphere surface for SERS performance for the detection of trace amounts of the antibiotic nitrofuranone (NFZ). Through the synergistic utilization of electromagnetic enhancement and charge transfer enhancement, the Raman signals of NFZ were significantly enhanced in the numerous hotspot regions with great intensity, a high EF of 2.31 × 10^11^, and an ultra-low detection limit of 5.80 × 10^−13^ M were achieved. Additionally, two-dimensional niobium telluride (NbTe_2_) nanosheets with ultrathin crystal morphology were prepared for the first time [164]. They used liquid-phase exfoliation and hydrothermal methods for the ultrasensitive detection of aromatic molecules and antibiotics. The NTNs were realized for the SERS detection and analysis of ciprofloxacin (CIP) and enrofloxacin (ENR) in the low concentration with LODs of 35.1 and 35.9 ppb, opening a new direction for the application of tellurium-based materials in food and drug safety detection. In addition, Zhang et al. [165] developed a TiO_2_/ZnO heterojunction with strong interfacial coupling and efficient carrier separation as a novel SERS substrate. The SERS enhancement of this highly active heterostructure is based on the substantial increase in photovoltaic electron utilization in the substrate, which also realizes the effective charge transfer in the substrate molecule system, which markedly improves the CT efficiency between the substrate and the molecule. This SERS substrate can be further applied to the ultrasensitive detection of egg antibiotic residues. The LOD of this substrate for enrofloxacin (ENR) residue in eggs was only 13.1 μg/kg (Figure 11f). It not only enables the sensitive and selective detection of individual antibiotic residues, but also offers the capability to achieve comprehensive screening of multi-component antibiotic mixtures present in animal-derived foods.

## 4. Summary and Outlook

As the study of non-noble nanomaterials progresses, the variety of substrates for surface Raman enhancement becomes more prosperous and diverse [166]. Non-noble metal materials with easy structure adjustment and compatibility advantages were greatly developed [167,168]. The need to design, synthesize and functionalize SERS nanosubstrates was summarized in recent years [169,170]. Since most non-noble metal SERS substrates are dominated by the CM effect of the material, non-noble metals also provide soil for mechanistic studies of chemical enhancement.

On the one hand, the enhancement effect of non-noble metal SERS substrates is generally low compared to that of noble metal substrates (Au and Ag), limiting their development and application. Currently, higher EFs are obtained by methods such as increasing the surface defects of the material at the cost of deteriorating the stability of the material, making it difficult to carry out subsequent applications. Therefore, the ability to combine the resonance conditions of the nanomaterials should be rationally adjusted, such as doping other materials such as noble metals [171] or developing new materials with higher stability, such as special micro-nanostructures or optical cavity structures [172]. Materials with increased sensitivity and good robustness are more useful in practical biomedical applications. To summarize, the linear range of detection of biomolecules for non-noble metal nanomaterials is in the range of 10^−5^–10^−12^, while the general detection range of noble metals is lower than that of non-noble metal substrates, being able to reach 10^−10^–10^−16^ M. We believe that this is due to the advantages of the EM mechanism itself for Raman enhancement. More important than LOD, however, is the ability to accurately predict concentrations over a range of concentrations that correlate with the actual concentrations likely to be encountered in the target sample. Therefore, the development of sensors should not be focused on low detection limits, but rather on matching practical applications, such as specific physiological concentrations in bioassays.

On the other hand, as an ideal medium for the study of chemical enhancement mechanisms, the study of the SERS mechanism in non-noble metal nanomaterials is still not perfect and comprehensive. Due to the many derivatives of different types of substrates with different chemical–physical properties and enhancement mechanisms, the principle of Raman enhancement of non-noble metals is yet to be thoroughly investigated. In addition, the enhancement effect of most non-noble metal SERS substrates is selective, and only a few molecules can be enhanced [173]. Therefore, a systematic refinement of the enhancement mechanisms of various non-noble metal nanomaterials is still needed to increase their exploitation rate. Most non-noble metal substrates are highly sensitive to charge transfer because of the enhanced chemical mechanism. It can be predicted that in the future, non-noble metal SERS substrates will have potential applications in building photovoltaic models and biosensing interfaces, the large-scale application of Raman enhancement of non-noble metals in clinical medicine still needs more exploration.

Moreover, most non-noble metals exhibit good biocompatibility and molecular selectivity, making them competitive in biomedicine [174]. Biocompatibility refers to the ability of a material to effectively reduce or avoid adverse reactions when in contact with biological tissues, to maintain normal biological function, and to promote tissue healing in the body. Titanium alloys, iron alloys, and some inorganic non-metallic compounds, among others, exhibit attenuated immune rejection and low toxicity when used as implants [175], making them a suitable choice for medical devices and implants. Nowadays, non-noble metal SERS substrates are becoming more and more sophisticated in practical biological fields, e.g., for preparing microfluidic chips and kits based on SERS nanomaterials, or for applications in clinical medicine and biomolecular detection technologies. However, as a matter of fact, the application of non-noble metals in biomedicine is still far from being mature enough for the noble metal system, and most of their detection limits and sensitivities are yet to reach the clinical indicators. Although showing their own advantages, they still need to be worked on.

Above all, more in-depth fundamental research and technology optimization are required to drive substantial progress in using non-noble metal nanomaterials in biomedical applications. Enhancing the safety, stability, and sustainability of materials is the key to ensuring that they are safe and effective in practical applications and meet the stringent standards for clinical applications. These materials may offer more cost-effective options than traditional noble metal-based implants and devices, broadening access to medical solutions [136]. As research continues to advance and breakthroughs are made, non-noble metal nanomaterials are expected to occupy an essential place in the future of medical devices and biosensors.

## Figures and Tables

**Table 1:** A summary of the research on the detection of SARS-CoV-2 by SERS technology in recent years.

SERS Substrates	Target Materials	Methodology or Platform	EF	LOD	References
Au nanoparticles	SARS-CoV-2	Anti-spike antibody attached gold nanoparticles	/	~4 pg/mL	[138]
Ag nanocubes with 4-MBA, 4-MPY, and 4-ATP		Assembled multiple surface receptors to induce molecules	1.4 × 10^10^	/	[139]
Au-Ag HNSs		Tri-mode lateral flow immunoassay (LFIA) platform	/	20 ng/mL	[140]
Au nanoplatelet film/MgF_2_/Au mirror/glass		A self-assembly approach to fabricate a large-area grating-like 2D plasmonic film.	2.0 × 10^5^	9.8 × 10^−11^ g/mL	[136]
Au GNC nanospheres		A core-gap shell-based nanostructure	/	39.65 fg/mL	[141]
Fe_3_O_4_@Au magnetic nanoparticles		A lateral flow immunoassay (LFIA)	/	1.43 pg/mL for prostate-specific antigen, 1.92 pg/mL for alpha-fetoprotein, 3.84 pg/mL for carcinoembryonic antigen	[142]
Fe_3_O_4_@Au MNPs		High-performance magnetic SERS tags for simultaneous ultrasensitive detection	2.61 × 10^6^	85 copies/mL for H1N1, 8 pg/mL for SARS-CoV-2, 8 pg/mL for RSV	[137]
Ag^MBA^@Au NP		A ligand-assisted epaxial growth method t	/	0.52 pg/mL	[143]
